# Evolution of the Immunoglobulin Isotypes—Variations of Biophysical Properties among Animal Classes

**DOI:** 10.3390/biom13050801

**Published:** 2023-05-08

**Authors:** Nancy D. Pomarici, Roberta Cacciato, Janik Kokot, Monica L. Fernández-Quintero, Klaus R. Liedl

**Affiliations:** Department of General, Inorganic and Theoretical Chemistry, Center for Molecular Biosciences Innsbruck (CMBI), University of Innsbruck, Innrain 80-82, A-6020 Innsbruck, Austria

**Keywords:** antibody isotypes, ig-like domains, evolution of immunoglobulins, coevolving residues

## Abstract

The adaptive immune system arose around 500 million years ago in jawed fish, and, since then, it has mediated the immune defense against pathogens in all vertebrates. Antibodies play a central role in the immune reaction, recognizing and attacking external invaders. During the evolutionary process, several immunoglobulin isotypes emerged, each having a characteristic structural organization and dedicated function. In this work, we investigate the evolution of the immunoglobulin isotypes, in order to highlight the relevant features that were preserved over time and the parts that, instead, mutated. The residues that are coupled in the evolution process are often involved in intra- or interdomain interactions, meaning that they are fundamental to maintaining the immunoglobulin fold and to ensuring interactions with other domains. The explosive growth of available sequences allows us to point out the evolutionary conserved residues and compare the biophysical properties among different animal classes and isotypes. Our study offers a general overview of the evolution of immunoglobulin isotypes and advances the knowledge of their characteristic biophysical properties, as a first step in guiding protein design from evolution.

## 1. Introduction

Antibodies or immunoglobulins (Igs) are specialized molecules that can recognize and bind foreign invaders. The antibody molecule is a Y-shaped glycoprotein, that can be present free in solution or bound to membranes. Immunoglobulins are formed by two identical light chains, including two domains each, and two identical heavy chains, usually with four or five domains each [1]. The N-terminal domain of each chain is a variable domain, and it is less conserved in sequence compared to the other domains, which are instead named constant The arms of the “Y”, the so-called antigen binding fragments (Fabs), are composed of two variable and two constant domains each [2]. The Fab directly recognizes and binds the antigen [3]. The rest of the constant domains form the crystallizable fragment (Fc), that plays a key role in mediating the effector functions, such as in the activation of the complement or in binding to Fc receptors [4,5,6].

The domains of the immunoglobulin chains share the common Ig-fold. They consist of approximately 70–110 amino acids, which form 7 β-strands in the constant domains or 9 in the variable ones, connected via loops and distributed in 2 antiparallel sheets [6,7]. In the constant domains, 4 β-strands (B, C, E and F) form a central common core, embedded in an antiparallel β-sheet sandwich with a total of 3 strands (A, D and G) [8]. The A, B, D and E strands shape the interface facing the opposite domain, whereas the C, F and G strands are exposed to the solvent or interact with the receptors. The variable domains have a similar configuration to the one found in the constant domains but with an opposite orientation. In fact, in this case, the strands A, B, D and E point towards the solvent and strands C, C’, C”, F and G face the opposite domain. In most Ig-like constant domains, the two sheets forming the sandwich-like structure are connected by a buried disulfide bridge between strands B and F, that highly stabilizes the structure [8,9,10]. In proximity of the disulfide bridge, a tryptophane in the β-strand C is highly conserved throughout the whole family [9]. 

Immunoglobulins exist in different isotypes, that differ mainly in the number of subunit domains and consequently in their function in the immune response. Only in mammals do five isotypes exist: IgA, IgM, IgG, IgE and IgD. They are distinguished by the types of heavy chain; γ, μ, α, δ and ε heavy chains characterize, respectively, IgG, IgM, IgA, IgD and IgE. The heavy chain sequences differ between isotypes in the number and position of disulfide bridges, in the number and types of oligosaccharides and constant domains and in their hinge region lengths [11]. In fact, IgM and IgE have a total of four heavy chain constant domains, thus one more than the other isotypes. The oligomerization state also changes between the different isotypes. Normally, the Igs are present as monomers, apart from the IgM, which usually exists as a pentamer, hexamer or tetramer in bony fish [12], and the IgA, which exists as a dimer or as a monomer [13]. The cryo-electron microscopy structures of mammalian secretory IgA and IgM, and dimeric IgA, revealed that a joining chain, folded together with the C-terminal peptide, is fundamental for the assembly of the polymeric Ig forms [14,15,16]. Interestingly, bony fishes lack the joining chain, suggesting that a different assembly mechanism characterizes this class of animals [17]. The Ig isotypes have a different distribution in the organism and are produced at different stages of the immune response [18]. While IgM is mainly produced in the primary immune response, IgG intervenes in the secondary immune response, activating the classical pathway of the complement system. IgA is the major antibody type in secretion, while IgE protects against parasites, causing allergic reactions [19,20]. Even if the function of IgD remains unclear up to now, previous works revealed that it may play an important role in lymphocyte differentiation [21] and, in addition, that secreted human IgDs are involved in innate immunity [22]. 

**Figure 1:**
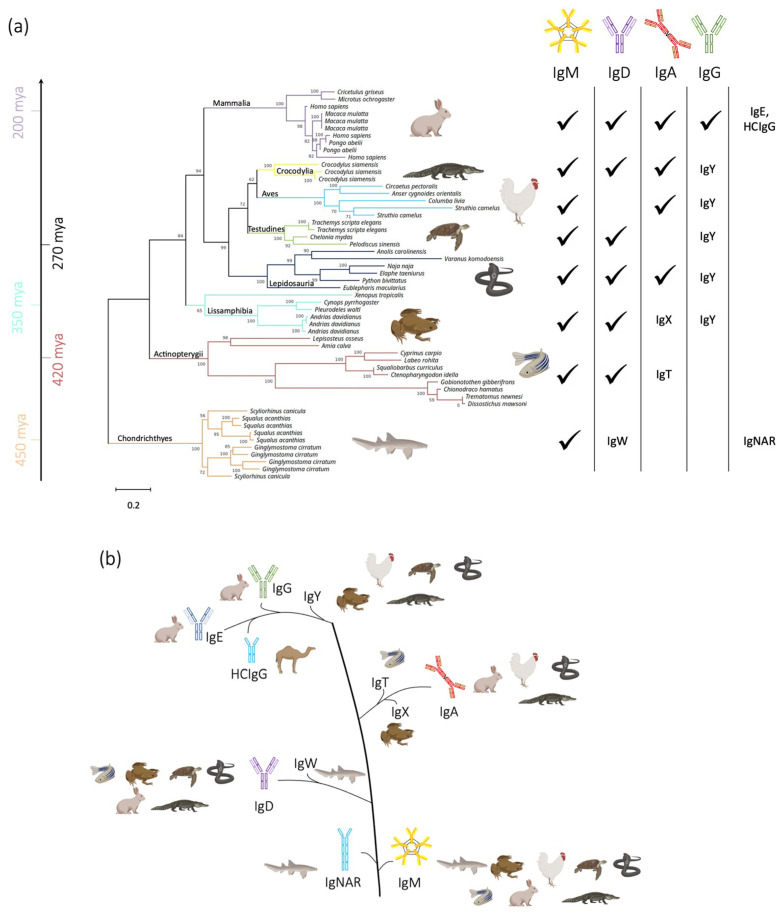
Phylogenetic analysis. (**a**) Phylogenetic tree of jawed vertebrates, constructed from the sequences of constant domains of IgM molecules. On the right side, a table illustrating the immunoglobulin isotypes present in each class of animal. The IgM molecule is schematically represented in its pentameric form, but it can exist also as a hexamer or as a tetramer in bony fishes. The tree is rooted on the Chondrichthyes branch. Mya: millions of years ago. (**b**) Schematic representation of the evolution of the immunoglobulin isotypes. The classes of animals that produce the specific immunoglobulin isotype are next to it.

From an evolutionary point of view, IgM is the most ancient antibody isotype [23], originated in cartilaginous fish (ca. 500 million years ago) and preserved in bony fish, amphibians, reptiles, birds and mammals, apart from coelacanths that lack this kind of immunoglobulin [24]. Starting from sequences of IgM constant domains, we reconstructed the phylogenetic tree of jawed vertebrates (Figure 1a), which is in line with the consolidated phylogenetic tree previously shown in the literature [25]. The cartilaginous fishes show only three different immunoglobulin types, namely IgM, IgW [26] and IgNAR [27]. IgW is orthologous to IgD, suggesting that not only IgM, but also IgD, are primordial types of immunoglobulin, that then perpetuate in most jawed vertebrates (Figure 1b) [28]. The IgNAR (new or nurse antigen receptor) instead is a characteristic heavy-chain-only antigen receptor [29]. Another case of a light-chain-deprived antibody is the HCIgG (heavy chain IgG) present in camelid [30]. IgG, IgE and IgA seem to have originated through the duplication of the IgM [31,32]. IgG and IgE are only present in mammals, but their orthologue, IgY, first found in birds [33], originated in amphibians [23,34]. IgA, instead, is first found in reptiles, but lower taxa already present the analogous isotype IgX (e.g., amphibians) [31,35,36]. The IgT isotype is found in bony fishes and it performs its functions mainly within the mucosal compartments [37,38,39].

In the last decades, evolutionary studies have become increasingly important in many fields, including 3D structure prediction of proteins or nucleic acids. By comparing the sequences of related proteins across different species, evolutionary relationships and functional constraints can be identified. In protein structures, for example, coevolving residues often correspond to intra- or interdomain contacts and this information can consequently be used to predict the structures of monomers or complexes, or even to predict the effects of mutations [40,41,42,43,44,45,46]. In this work, we focus on the constant domains of immunoglobulins, and we compare the biophysical properties of Igs among different isotypes and animal classes, to understand how they evolved over time. This study provides a broad overview of the mutations and modifications that nature allowed in the immunoglobulin domains, highlighting the residue with a high likelihood to be mutated and the ones that, instead, are conserved and therefore fundamental to keep the correct fold. 

## 2. Materials and Methods

### 2.1. Dataset Generation and Alignment

The protein sequences for each immunoglobulin isotype have been collected via protein BLAST (basic local alignment search tool) search [47]. A separate search was performed for each animal class. The sequences were searched in the non-redundant protein sequences (nr) database, a database introduced by NCBI to reduce the redundancy of the available information. The expected threshold (E_value_) was set to 0.01 to select a significant alignment. The remaining parameters were set to default values. The sequences with 100% identity and the same length as the reference were excluded. After the BLAST search, the complete sequences were downloaded.

All the sequences obtained from the BLAST search were merged and aligned using Clustal Omega [48] implementation in Unipro UGENE [49]. Clustal Omega is a powerful multiple sequence alignment program that uses seeded guide trees and profile hidden Markov models (HMMs) to generate robust alignments [50]. We used trimAl [51] to remove the positions in the alignment with gaps in 80% or more of the sequences. After aligning the complete sequences, we separated and labelled the single domains, removing identical sequences in each subset. The final number of sequences in the dataset is shown in Table S1. The complete list of the accession codes, that can be accessed via the Uniprot/Uniparc database [52], is also provided in the Supplementary Materials. In this work, we numbered the sequences from 1 to 117, including gaps. In addition, we provide in Figure S1 the IMGT alignment and numeration for a representative C_H_1, C_H_3 and C_L_ domain output of the IMGT webserver [53,54,55,56]. The complete alignment of all domains is also provided as Supplementary Materials. We then evaluated the sequence conservation of the multiple sequence alignment, using the script developed by Capra et al., based on Jensen–Shannon divergence [57].

### 2.2. Phylogenetic Tree

Starting from the multiple sequence alignment of the complete constant domains sequences of IgM molecules, we generated the phylogenetic tree using the software IQ-TREE 2 [58], which uses the maximum likelihood method to estimate the tree connections [59]. We chose the IgM constant domains sequences because IgM is a primordial Ig isotype and therefore is present in all jawed vertebrates [23]. Before performing the phylogenetic analysis with the maximum likelihood method, a search of the best amino acid substitution model was performed using the software MEGA X version 10 (Molecular Evolutionary Genetics Analysis) [60]. In our case, the WAG model [61] obtained the lowest BIC (Bayesian information criterion) score [62], and therefore is considered to better describe the substitution pattern. In addition, we applied a discrete gamma distribution with 4 categories to take into account the influence of rate heterogeneity across sites [63]. For the statistical evaluation of the tree, the ultra-fast bootstrapping (UFBoot) is set to 1000 to judge the statistical support of the relationship inferred [64,65]. 

### 2.3. Evolutionary Coupled Residues Analysis

For the analysis of the evolutionary coupled residues, we used the EVcouplings webserver [40]. The C_H_3 domain of a human IgG was used as reference structure (PDB: 3AVE [66]). The server performed a homology search in the Uniref90 database [67] to find similar sequences to the target. A Bitscore of 0.3 was used as sequence inclusion threshold for the alignment and a maximum of 5 search iterations was allowed to generate the alignment. A maximum of 20% gaps was accepted (position filter: 80%) and we only retained sequences that covered at least 80% of the length of the reference structure. After the sequence search, redundant sequences with a sequence identity >90% were discarded and the sequences with a pairwise sequence identity higher than 80% were clustered, to decrease the influence of highly redundant sequences in the statistical inference. Finally, the pseudo-likelihood maximization was used as a statistical inference method [68]. We downloaded the final result of the online calculation and analyzed it using the EVcouplings python framework [69]. The contact maps were constructed considering coevolving pairs with a probability >0.8. Only long-range interactions were considered (the paired residues are at least 4 residues’ distance apart in the sequence). In order to evaluate if these pairs also form intradomain contacts, we used the same reference as in the initial search: the monomeric C_H_3 domain of a human IgG (PDB: 3AVE). The residues were considered in contact if their minimum atom distance in the reference structure is lower than or equal to 5 Å. The same distance criterion was also used to evaluate the interdomain contacts and, in addition, the GetContacts software provided the interaction type information [70]. In order to look at pairs that may be involved in interdomain contacts, we considered all the coevolving pairs with a probability higher than 0.6.

### 2.4. Sequence Logos

The protein sequences were illustrated via sequence logos, which gave an idea of the occurrence of each residue in each position and, consequently, of its biochemical properties. The sequence logos were created with the python package Logomaker [71]. We used the color scheme ‘chemistry’, generated in the same python package, that partially describes the amino acids properties: apolar residues (A, I, F, L, M, P, V, W) in black, polar residues (C, G, S, T, Y) in green, polar amides (Q, N) in purple, acidic residues (D, E) in red and basic ones (R, K, H) in blue.

### 2.5. Principal Component Analysis (PCA)

The PCA analysis was used to lower the dimensionality of the sequence space and visualize separations between different animal classes and/or immunoglobulin isotypes. The analysis was performed in Python 3 [72]. First, the sequences of the dataset were encoded using the One Hot encoding. Apart from the 20 naturally occurring amino acids, additional characters were present in the sequences: ‘-‘ for the gaps, ‘B’ for any undetermined asparagine or aspartic acid, ‘X’ for any not defined amino acid and ‘Z’ for any undetermined glutamine or glutamic acid. Therefore, the final matrix obtained after encoding has a size of 117 (length of the sequences with gaps) × 24. Then, the PCA analysis was performed using the implementation in Scikit-learn [73]. We then assigned a value to each letter in the alignment, representing some biophysical properties. The Wimley–White scale was used to calculate hydrophobicity [74], the normalized van der Waals volume was determined to represent the residue size [75], and we assigned 1 to Asp, Glu, Arg and Lys to indicate the presence of a net charge. For the other amino acids, an average of the values of each property was made (for B, average of the properties of Asn and Asp, for Z, average of the properties of Gln and Glu and for X, average between all amino acids).

## 3. Results

In this work, we analyzed the biophysical properties of immunoglobulin isotypes and how they evolved in different animal classes. We focused on the constant domains of immunoglobulins because of their highly conserved structure and their critical role in the immune response [76]. First, we considered the coevolving residues in the monomers since they play an important role in maintaining the correct protein fold. However, not all the evolutionary coupled residues form intradomain contacts, but some are instead involved in interdomain interactions. These residues are fundamental in the dimerization process and in the interaction with other receptors. In addition, we analyzed the C_H_1, C_H_3 and C_L_ domains belonging to different isotypes and animal classes, highlighting differences in biophysical properties that explain the observed separation in the PCA space. 

### 3.1. Evolutionary Couplings Analysis

**Figure 2:**
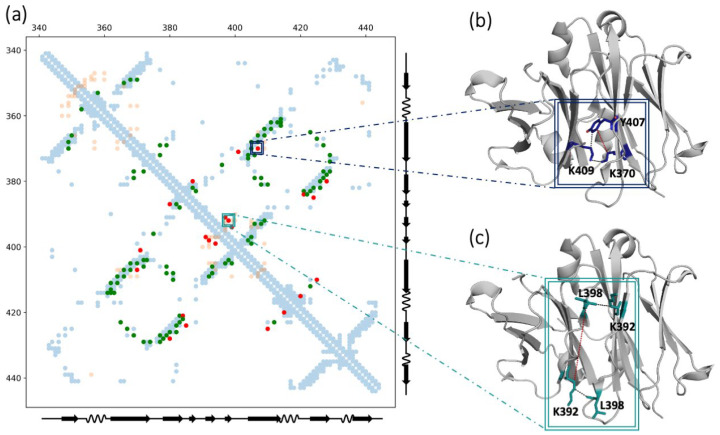
Evolutionary coupling analysis. (**a**) Contact map of the evolutionary coupled residues. The residue pairs that are predicted to be coupled in evolution and form an intradomain contact are shown in green. The residue pairs that are predicted to be coupled in the evolution but do not form intradomain contacts are depicted in red. In light blue, the intradomain contacts present in the reference structure (PDB: 3AVE) are illustrated. In light orange, the interdomain contacts of the same reference structure are shown. The numeration and the secondary structure assignment are inferred from the same PDB reference structure. The coupled pairs that do not form intradomain contacts but are involved in interdomain interactions are highlighted in squares. (**b**) Structural representation of the coevolving pair Y407-K370 (IMGT: Y86-K26). They do not directly interact with each other in the monomer structure, but they are both close to the same residue (K409, IMGT: K88) on the opposite domain. (**c**) Structural representation of the coevolving pair L398-K392 (IMGT: L84.1-K79). They do not directly interact in the monomer structure, but they make an interdomain contact with each other.

We performed EVcouplings analysis using the webserver tool [40], as described in the Section 2. The resulting contact map is shown in Figure 2a. We used the numeration of the C_H_3 reference domain (PDB: 3AVE). We chose the C_H_3 domain as reference because of its presence in every isotype, its relevant role for the production of bispecific antibodies [77] and its common fold. In particular, the C_H_3 domain is the only domain that can homodimerize in common IgGs, therefore also interdomain interactions can be evaluated. A total of 82% of the evolutionary coupled residues form an intradomain contact (green dots), meaning that these residues are relevant to maintain the correct fold. However, some other pairs are separated in the structure with a minimum atom distance higher than our cutoff (5 Å), and therefore are not considered to make intradomain interactions in our reference structure (red points in Figure 2a). Nevertheless, these residues can still be relevant for the overall protein fold, by making interactions with neighboring domains or with other partner molecules. For example, L398 and K392 (IMGT: L84.1 and K79) are predicted to be coupled but they are located on opposite sides of the D-strand, and are consequently too far apart to interact (Figure 2c). However, the same residues make an interdomain contact with each other, which can explain why they evolved together. In other cases, there may not be any significant intra- or interdomain connections among the coevolving residues, but their evolutionary correlation can be explained by their interaction with a common partner. This is the case for K370 and Y407 (IMGT: K26 and Y86), which are predicted to be evolutionary coupled but are separated in the reference monomer. Looking at the dimeric interactions, both residues are at a close distance from the same amino acid (K409, IMGT: K88) in the opposite domain (Figure 2b), which can explain their coevolution. 

### 3.2. Evolution-Based Analysis of Interdomain Contacts

**Figure 3:**
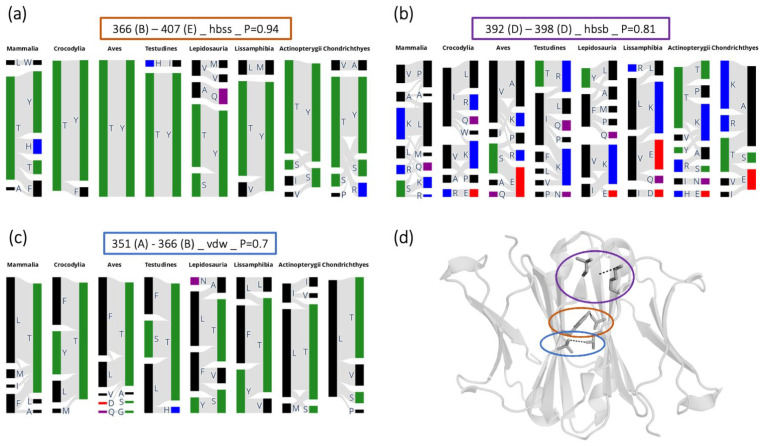
Evolutionary analysis of interdomain contacts. (**a**–**c**) Plots showing the residue pairs present in the two coupled positions. The shown residue pairs are only those that occur in more than 2% of the total number of sequences within each animal class. The colors represent the biochemical property of the residue and are in line with the sequence logos color-coding. The title of each plot includes the residue positions (IMGT: (**a**) 22–86, (**b**) 79–84.1, (**c**) 7–22), the β-strand/loop where they are in the structure in brackets, the interaction type that the two residues make in the reference structure and the probability obtained from the EVcouplings analysis. (**d**) The evolutionary coupled residues that make interdomain contacts in the reference C_H_3–C_H_3 interface (PDB: 3AVE) are shown as sticks. The circle around each pair is color-coded according to the boxes in (**a**–**c**).

As functional Ig molecules are usually formed by multiple chains that dimerize, it is also important to study the interactions that are crucial for chain pairing. Therefore, we studied which of the coevolving residue pairs, resulting from the EVcouplings analysis [40,69], correspond to an interdomain contact in the C_H_3–C_H_3 interface. The type of contact that they make is assigned with the GetContacts software [70]. Many of the interdomain interactions are excluded from our set of coevolving residue pairs, because we are only considering long-range interactions (between residues that are more than four residues apart in the sequence). To better visualize which mutations are allowed, we looked at the residues that are present in the evolutionary coupled positions in the different animal classes (Figure 3). In some cases, evolution kept the properties of specific residues highly conserved, as in positions 366–407 (IMGT: 22–86) (Figure 3a) or in positions 351–366 (IMGT: 7–22) (Figure 3c). Sometimes, one residue that is part of the pair can be modified, changing the biophysical properties and probably also the interaction type in the pair, e.g., the highly conserved Y407 (IMGT: Y86) can be substituted by an H in mammals or by an R in sharks (Figure 3a), or a polar residue can be found in position 351 (IMGT: 7) (Figure 3c), probably forming hydrogen bonds. Some pairs are instead highly variable, such as positions 392–398 (IMGT: 79–84.1) (Figure 3b). In this case, the side chain-backbone hydrogen bond between a charged residue and an apolar one is recurrent in animal classes that are separated by evolution (Chondrichthyes and Mammalia) and, sometimes, the residue’s properties are inverted between the two positions (in Lissamphibia, Lepidosauria, Testudines and Crocodylia, 398 (IMGT: 84.1) is the charged residues and 392 (IMGT: 79) the apolar one). Other combinations are also possible, e.g., two apolar residues or one polar and one charged residue, suggesting that this residue pair is highly variable. The most common mammalian residues in the investigated pairs are depicted as sticks in a representative C_H_3-C_H_3 dimer in Figure 3d, to better visualize their location in the interface. More examples of coevolving residues that are involved in interface contacts are shown in Figure S2. 

### 3.3. Sequence Conservation

**Figure 4:**
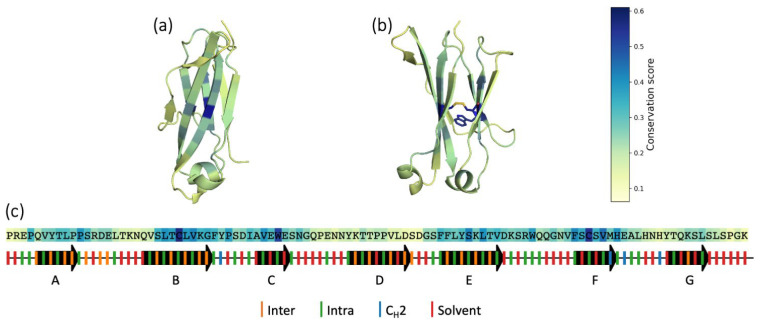
Sequence conservation. (**a**,**b**) Frontal (interface-oriented) and lateral view of a C_H_3 domain, in which each residue is colored according to its conservation score. (**c**) Representative C_H_3 domain sequence (PDB: 3AVE), in which each residue is colored according to the conservation score. The secondary structure is represented below the sequence: the strands are shown as arrows and the loops as lines. The ticks on the secondary structure represent the direction where the residues point: green if they point in between the β-sheets intradomain, orange towards the facing domain, blue towards the C_H_2 domain and red towards the solvent.

From the multiple sequence alignment of the sequences in our dataset, we could estimate the conservation of each residue position, as described in the Section 2. As expected, the two cysteines in strands B and F, and tryptophane in strand C, show a high conservation score (Figure 4). In general, a high degree of conservation is present in strands B, C, E and F, which are the ones that shape the central core of the structure. Instead, the strands A, D and G, and the loops, show higher variability and are less preserved during evolution. The most conserved residues are the ones that point inside the two β-sheets and that contribute to the stability of the fold (Table S2). The residues that make interdomain interactions are instead more variable (Table S2), meaning that different types of contacts are allowed at the interface between two domains. 

### 3.4. C_H_3 Domains: Biophysical Properties in Different Isotypes and Animal Classes

We systematically compared the biophysical properties of sequences originating from different isotypes and animal classes. For the analysis of the C_H_3 domains, we selected the third constant domains of each isotype, but the C_H_4 for the IgY, IgM and IgE. In fact, these isotypes share a high degree of similarity and they all contain an additional constant domain [78]. It has been shown that C_H_1, C_H_2 and C_H_3 of IgG correspond to the Cµ1, Cµ3, and Cµ4 of IgM, respectively, whereas the hinge region of IgG corresponds to the Cµ2 of IgM [79]. We refer to each isotype with the nomenclature used in mammals (IgG, IgM, IgE, IgD and IgA), but we also include in each subgroup the isotypes present in the other animal classes: IgG also includes IgY, IgA also comprises amphibians’ IgX and IgT in bony fishes and IgD includes the IgW present in sharks. Here, we are using a different numeration compared to the one used for the EVcouplings analysis: the residues are numbered from 1 to 117, including gaps. A translation between the two numerations for a reference sequence (C_H_3 domain of 3AVE) is shown in Figure S3. In addition, the IMGT numbering obtained from the webserver for the same reference sequence is also shown [53,55,56].

First, we performed a PCA analysis of the encoded sequences (see Section 2), to observe if we could find any separation between isotypes and animal classes, based only on the sequence information. Figure S4a,b shows the PCA space of the C_H_3 domain sequences colored according to isotypes and animal classes, respectively. The first 4 PCs can only explain 28.6% of the total variance in the dataset (PC1: 11.8%, PC2: 6.1%, PC3: 5.7% and PC4: 5%), meaning that our sequences cover a noisy and multidimensional space that is hard to completely describe with this technique alone. However, already the first four PCs can separate various isotypes: PC1 separates the mammalian IgG, while PC2 can distinguish between IgM and IgE. PC4 distinguishes the mammalian IgA from the other sequences. To understand which biochemical properties can drive this separation, we plotted the normalized charge, hydrophobicity and van der Waals volume values on the same PCA space. In order to normalize the properties, we assigned a value representing the considered property to each residue of the sequence, summed up the values in the sequence, and divided by the number of positions that are not gaps. For the C_H_3 domains, both charges and hydrophobicity play a role in the isotypes‘ separation in the PCA space. In fact, IgG and IgE seem to have higher normalized charge values compared to the remaining isotypes (Figure S4c), and consequently a lower hydrophobicity (Figure S4d).

**Figure 5:**
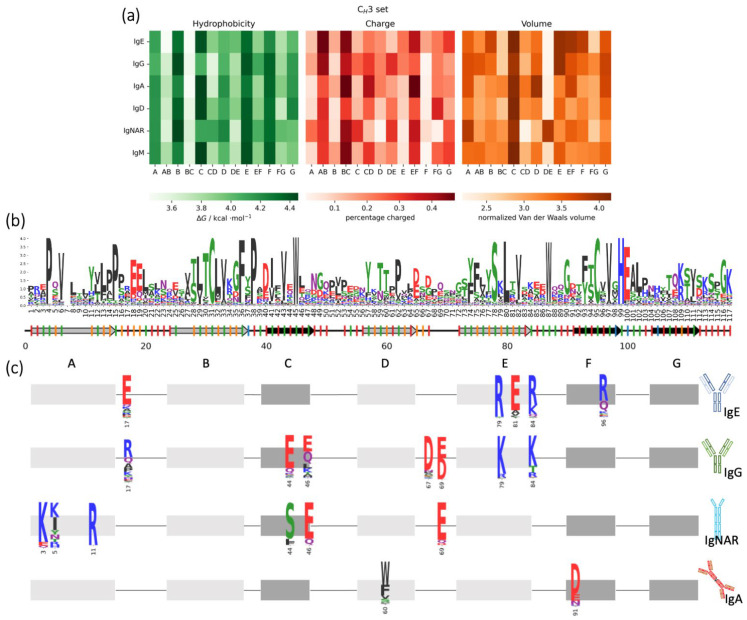
Biophysical properties of C_H_3 domains between different Ig isotypes. (**a**) Heatmap showing the occurrence of the biophysical property (hydrophobicity, charge and volume) in each secondary structure element of the Ig isotypes. (**b**) Sequence logo made from the alignment of all the C_H_3 domains sequences. Under the logo, a representation of the secondary structure of an exemplary C_H_3 domain; the light grey arrows represent the strands that point towards the facing domain, instead the black ones are the strands that point towards the solvent; the lines are the loops. The ticks on the secondary structure show where the residue points: if red, it points towards the solvent, green, inside the β-sheets, orange, in the interface with the facing domain, and blue, towards the C_H_2. A missing tick means that the reference structure presents a gap in that position. (**c**) Schematic representation of the residues in position 3, 5, 11, 17, 44, 46, 60, 67, 69, 79, 81, 84, 91 and 96 (IMGT: 1, 3, 5, 11, 40, 42, 81, 84.4, 84.6, 88, 90, 93 and 105, respectively), showing relevant differences between isotypes.

To evaluate which residue positions are responsible for the differences among isotypes and animal classes, we averaged the biophysical properties of the residues at each position in the sequence (Figure S5a). For a more general overview, an average of the properties between the positions belonging to the same secondary structure element is presented in Figure 5a. The annotation of the secondary structure is based on a reference PDB structure (3AVE). There are some secondary structure elements that stay conserved between the different isotypes, for example the loop BC contains many negatively charged residues, and the strand B is usually highly hydrophobic (Figure S5a,b). Also, the loop AB is heavily charged, mainly due to the presence of two negatively charged residues in position 18 and 19 (IMGT: 12 and 13), pointing towards the interface with the opposite domain. IgG and IgE show an even higher overall charge of the AB-turn, thanks to an additional charge (mostly positive for the IgG and negative for the IgE) in position 17 (IMGT: 11) (Figure 5c). The strand A, in the interface with the opposite domain, is characterized by a relatively high hydrophobicity, caused by the presence of many P, V or L, but the IgNARs show a higher number of charged residues compared to the rest of the isotypes. In fact, the residues in position 3, 5 and 11 (IMGT: 1, 3 and 5) are highly populated with positive charges. Strand C is characterized by a large van der Waals volume, mainly dominated by the highly conserved W in position 45 (IMGT: 41) (Figure 5b). However, the overall hydrophobicity of strand C of IgG and IgNAR is lower compared to that of the other isotypes, because of the presence of charged or polar residues in position 44 and 46 (IMGT: 40 and 42), where other Ig types instead have hydrophobic residues. Strand D is conserved in hydrophobicity and van der Waals volume, except for IgA that, in position 60 (IMGT: 81), presents bulky residues as F or W (Figure 5c). Interestingly, this residue is normally involved in interdomain interactions with the residue in the same position in the opposite domain, meaning that, in the presence of aromatic residues, as in IgA, pi stacking interactions are possible. The DE-turn is usually quite charged, but the highest number of charged residues is present in the IgG and IgNAR, with a prevalence of negative charges in position 67 and 69 (IMGT: 84.4 and 84.6). On the other side, the E-strand typically has a limited number of charged residues. However, in IgG and IgE antibodies, charges are prevalent, particularly in locations 79, 81, and 84 (IMGT: 88, 90 and 93), where they can interact with the facing domain. Other interesting changes in charges happen in the EF-turn, where IgA presents a highly occurrent negative residue in position 91 (IMGT: 100), while in position 96 (IMGT: 105) (F strand) the IgE is characterized by a positively charged amino acid. This analysis is perfectly in line with the previous PCA result, highlighting a higher charge in the IgG and IgE, with consequent lower hydrophobicity, and a similar trend in the IgNAR molecules, that were instead not distinguishable in the PCA space.

The biophysical properties of C_H_3 domains show fewer differences among animal classes (Figure S6). The strands are in general more hydrophobic than the loops, which are instead characterized by a higher number of charged residues. Strand C has the highest hydrophobicity level, due to the conserved W45 (IMGT: W41). Furthermore, the A and B strands are hydrophobic, with no major differences among animal classes. Strands E and F, even if highly hydrophobic, show some small differences; in fact, sharks and bony fishes reveal a lower charge compared to the remaining animal classes, since the positive charge in position 79 (IMGT: 88) (E strand) is substituted respectively by a L or a Q. On the other hand, the average van der Waals volume of the F strand in bony fishes is higher, because of the Y98 (IMGT: Y107) (F strand) that replaces the smaller G. The D strand is less hydrophobic than the others, apart from the bony fishes, in which the W63 (IMGT: W84) increases the overall strand hydrophobicity. On the contrary, the loops present more charged residues. The Lepidosauria show a higher charge level than the other classes in the DE-turn, because of positively and negatively charged residues, respectively, in positions 67 and 72 (IMGT: 84.4 and 85.4).

### 3.5. C_H_1 Domains: Biophysical Properties in Different Isotypes and Animal Classes

For the analysis of the C_H_1 domains, we selected the first domains from the aligned sequences retrieved after the BLAST search, we encoded them and performed a PCA analysis (Figure S7). In this case, the first four PCs show a good separation of the Ig isotypes and animal classes, even if the percentage of variance explained is low (PC1: 10.7%, PC2: 6.4%, PC3: 6.3%, PC4: 4.6%). PC1 clusters the IgG and IgE together, while PC2 separates the IgD from the IgM. The IgAs are clearly distinguished from the other isotypes, thanks to the separation in PC3. Plotting the normalized biophysical properties in the same PCA space, IgG and IgE show low charge values and van der Waals volumes compared to the other isotypes (Figure S7c,d), and the same low charge values can be spotted in the IgA sequences (Figure S7e).

In addition, we analyzed the residue properties that different isotypes and animal classes have at each position, to find out which modifications evolution allowed. The heatmap in Figure S8a shows the properties of the secondary structure elements in various isotypes. The secondary structure was inferred from a reference PDB structure (PDB code: 5DK3 [80]). In general, the strands show a conserved hydrophobicity between different Ig isotypes, compared to the loops. Few differences are visible in the D and G strands. The D strand is highly hydrophobic, mainly because of the conserved P61(IMGT: P82); however, IgD and IgM show a higher overall charge in this strand since they are characterized by a positively charged residue in position 58 and 59 (IgD only) (IMGT: 79 and 80) (Figure S8c). The G strand, instead, is much less hydrophobic than the other strands and, especially in IgG and IgNAR, it has abundant charged residues in positions 108, 109, 110 and, for IgG, also in position 106 (IMGT: 118, 119, 120 and 116). The K in position 109 (IMGT: 119) is particularly relevant because it is involved in a frequently occurring interdomain salt bridge in mammalian IgG; interestingly, this charge is not so conserved in the other isotypes. The loops, normally more charged, show a higher variability. The IgNARs usually have more charged residues than the other isotypes: this is true for the AB-turn (positions 18, 19, 21, IMGT: 12, 13, 15), in position 39 in the BC-loop (IMGT: 35), and in the DE-turn (in position 68 and 71, IMGT: 84.5 and 85.5). The other isotypes generally show fewer differences. Some interesting properties are the two highly hydrophobic residues in positions 37 and 39 in the IgA (IMGT: 29 and 35), neighbors of the conserved hydrophobic P38 (IMGT: P30), which enlarge the hydrophobic property of the BC-loop of IgA. The F37 (IMGT: F29) that characterizes the IgA can also be found in other isotypes, but is instead missing in IgD and IgM that, in the same position, have a smaller residue (L or Q). 

**Figure 6:**
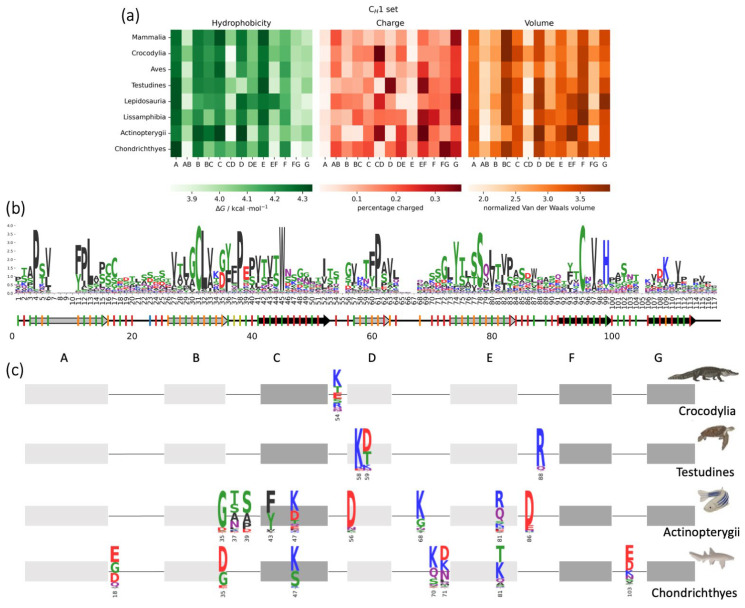
Biophysical properties of C_H_1 domains between different animal classes. (**a**) Heatmap showing the occurrence of the biophysical property (hydrophobicity, charge and volume) in each secondary structure element for the different animal classes. (**b**) Sequence logo made from the alignment of all the C_H_1 domains sequences. Under the logo, a representation of the secondary structure of an exemplary C_H_1 domain: the light grey arrows represent the strands that point towards the facing domain, while the black ones are the strands that point towards the solvent; the lines are the loops. The ticks on the secondary structure show where the residue points: if red, it points towards the solvent, green, in between the β-sheets, orange, in the interface with the facing domain, blue, towards the C_H_2 and yellow, towards the variable domain; a missing tick means that the reference structure presents a gap in that position. (**c**) Schematic representation of the residues in position 18, 35, 37, 39, 43, 47, 54, 56, 58, 59, 68, 70, 71, 81, 86, 88 and 103 (IMGT: 12, 27, 29, 35, 39, 43, 45.3, 77, 79, 80, 84.5, 85.6, 85.5, 90, 95 and 97, respectively), showing relevant differences between animal classes.

The animal classes show major differences in the biophysical properties of the C_H_1 domains, mainly characterizing bony fishes and sharks. A sequence logo obtained from all the C_H_1 domain sequences in our dataset is depicted in Figure 6b. The changes in properties in the Chondrichthyes often reflect those of the IgNAR. In fact, the sharks’ C_H_1 domains are characterized by a higher number of charged residues in the AB and DE-turns, caused mainly by positions 18, 70 and 71 (IMGT: 12, 85.6 and 85.5) (Figure 6c), as in the IgNARs. Moreover, sharks also have additional charges in the B strand (position 35, IMGT: 27), C strand (position 47, IMGT: 43), E strand (position 81, IMGT: 90) and FG-loop (position 103, IMGT: 113). It is important to notice that the charged residues in the B and E strands are interdomain interface residues and therefore they probably allow additional salt bridges. The increase in charged residues, highly common for sharks, is also visible in some regions of the C_H_1 domains of the bony fishes: C and E strands (position 47 and 81, respectively, IMGT: 43 and 90), and CD, DE and EF-turns (position 56, 68 and 86, respectively, IMGT: 77, 84.5 and 95). Furthermore, in this case, the additional charges in the E-strand and DE-turn could interact with the facing domain, making additional salt bridges. However, the first part of the sequence of the C_H_1 domains of bony fishes is characterized by a loss of charge (position 35 and 39, IMGT: 27 and 35, B strand and BC-loop, respectively), and a consequent increase in hydrophobicity and van der Waals volume in the C strand, for the recurrent F or Y residues in position 43 (IMGT: 39). The charged residue count appears to be the primary distinguishing factor, as depicted in the heatmap in Figure 6a. This holds true not only for sharks and bony fishes but also Crocodylia, whose charge percentage in the BC-loop is highly increased by the residue in position 54 (IMGT: 45.3), and in the Testudines. In the turtles, in fact, the D strand and the EF-turn appear to have a higher charge percentage, and this is mainly due to residues 58 and 59 in the D strand and 88 in the EF-turn (IMGT: 79, 80 and 97, respectively).

### 3.6. C_L_ Domains: Biophysical Properties in Different Isotypes and Animal Classes

Antibody light chains can exist as four isotypes: lambda (λ), kappa (κ), sigma and sigma-cart. However, the sigma isotype is only present in ectothermic vertebrates and sigma-cart in sharks only [81]. In this analysis, we focus on Igκ and Igλ, which are instead present in all vertebrates. The first principal component in a PCA analysis, even explaining only 17.1% of the total variance, can distinguish between these two mammalian isotypes (Figure S10), while the other animal classes tend to exhibit more overlapping characteristics. 

There are only few charged residues that distinguish the two isotypes, and they are mainly located in the BC and DE-turn and in the G strand (Figure S11b,c). Igκ is more charged in the loops (position 39 and 40 in the BC-loop and position 68 in the DE, IMGT: 35, 36 and 84.5, respectively), while Igλ has more recurrent charges in position 107 and 108 in the G strand (IMGT: 117 and 118). Hydrophobicity and van der Waals volume are instead conserved between the isotypes. The higher charge of the Igκ molecules is confirmed by the PCA plots in Figure S10c.

More differences are present between the animal classes and, also in this case, they mainly concern charges. The strands stay again more conserved compared to the loops, even if the hydrophobicity of strands C and F in sharks is lowered by the presence of some charges (position 42 and 44 in strand C and position 98 in strand F, IMGT: 38, 40 and 107). This difference in charge can also be spotted in the CD (position 52) and DE-turn (position 69, IMGT: 84.6). The few other species that show a higher charge in the loops are highlighted in Figure S12b,c.

## 4. Discussion

In this study, we systematically investigated the amino acid composition of the immunoglobulin isotypes in different animal classes, to gather insights into the substitutions that have naturally occurred within these proteins. Immunoglobulins are key components of the jawed vertebrate adaptive immune response. Since their origin over 500 million years ago, they have widely differentiated during vertebrate evolution resulting in a diverse repertoire of Ig isotypes [23,36]. The antibody isotype is determined by the heavy chain constant region, which is important for determining its effector function and binding to cell surface receptors. The ability to express different isotypes evolved early, since even the most primitive jawed vertebrates, cartilaginous fish (e.g., sharks), have multiple constant heavy chain genes [82]. The distribution of the Ig isotypes in the different animal classes is shown in Figure 1. We gathered sequences belonging to each isotype and animal class and performed a thorough analysis of the conserved residue positions and the ones that instead allow changes in biophysical properties. We believe that a strong synergy exists between molecular evolution and protein biophysics. Thus, our goal is to collect evolutionary data that will aid in the comprehension of how natural selection improved protein design to produce functional molecules.

Evolutionary studies play a crucial role in understanding how individual amino acids contribute to the structure and function of a protein of interest [83,84]. The proper 3D fold of a protein structure is essential for its function, and evolutionary pressure imposes strict limitations on the variability of amino acid sequences among related proteins. A specific alteration in one position may necessitate certain changes in the residue it interacts with, in order to maintain the protein’s structure and functionality. As a result, there can be associations between the amino acid compositions at different positions in the protein sequence [85]. This insight has been central to progress in computational structure prediction starting from classical methods [40,42,86,87], to achieve the current high-accuracy with the introduction of deep learning [88]. Following the same line, we performed an analysis of the coevolving residues in the Ig constant domains. The focus on the constant domains is driven by their central role in protein engineering to obtain better-performing therapeutics [77,89,90] and because of their highly conserved fold [8]. From the EVcouplings analysis, we found that 82% of the coevolving residues form intradomain interactions in our reference C_H_3 domain structure (PDB: 3AVE), which is in line with the established knowledge that evolution constrains the amino acid sequence variations to maintain the protein structure and function [42,85,86]. For the small fraction of coevolving pairs that are located far away according to our reference crystal structure, we investigated various biological reasons for the appearance of such long-distance direct correlations. In our case, two coevolving but distant residues are involved in an interdomain interaction with each other. Another possibility is that the paired residues, even if do not interact directly with each other, instead make contact with the same partner residue in the opposite domain. Some other coupled residue pairs are involved in both intra- and interdomain interactions within the homodimer. These insights highlight the importance of interdomain interactions in the immunoglobulin assembly process and their contribution to the overall structure stability [91,92]. We can hypothesize that the few remaining coupled residues that are separated by more than 5 Å in our reference protein structure can be in contact in at least one homologous structure, as shown by Anishchenko et al. [93]. 

Our dataset, obtained via pBLAST search, groups the various isotypes in all the vertebrate classes. Therefore, we combined the coevolutionary information obtained from the EVcouplings webserver with biophysical property information derived from the amino acid sequence. While some residue pairs remain fixed in their properties, allowing the same type of interaction between them, others exhibit greater variability, such as the coupled residue 392–398 (58–64 in the alignment numeration, IMGT: 79–84.1), both situated in the D strand. Notably, the charged–apolar residue combination is the most prevalent; however, their placement is not consistent, resulting in the possibility of the charge being in position 392 (IMGT: 79) and the apolar residue in position 398 (IMGT: 84.1), or vice versa. This interchange in biophysical properties serves as a clear indication of coevolution between the residues. 

A conservation analysis based on the sequence alignment shows that, as expected, the most conserved residues are the cysteines in the B and F strands and the tryptophane in the C strand in close proximity [94]. The intradomain disulfide bond, even if highly conserved, is not required for folding and association [8]. However, the thermodynamic stability of the reduced C_H_3 dimer is much lower than that of the oxidized state [95]. We find that the β-strands are much more conserved than the loops connecting them, especially B, C, E and F which shape the core of the structure. By comparing conservation scores across residue positions, we can deduce that residues pointing in the β-sheets are better preserved than those directed towards the solvent. The interdomain residues are ranked in between, indicating that contact types within the dimer exhibit more variation compared to contacts critical to the monomer folding. 

Furthermore, a comprehensive investigation on the biophysical properties of individual residues was conducted, to determine the distinctions between various isotypes and animal classes. In general, sharks, which are the first animal class that developed an adaptive immune response, have more charges throughout the overall sequence of each considered constant domain. In fact, the C_H_3 domains of IgNARs contain positively charged residues in the A strand, which usually has a hydrophobic character, and positive ones in the C strand and the DE-turn. Furthermore, the C_H_1 domains of the IgNARs follow the same trend, showing predominant charged residues in various loops and strands. These characteristics contribute to the peculiar properties of the IgNAR molecules. In fact, contrary to the IgG C_H_1 domain which is unfolded in isolation [96], IgNAR C_H_1 and C_H_3 form a dimer both in crystal structure and in solution, with C_H_1 dimerization driving the formation of the C_H_3 dimer [97]. Moreover, the IgNAR C_H_1 dimer also differs from the others because of the wide angle between the two domains, which is fundamental to ensuring an appropriate spatial separation of the variable domains [39,97]. The peculiar biophysical properties of IgNARs are then reflected in the C_H_1 domains of sharks and hold true for the C_L_ domains. As vertebrate evolution proceeds, the prevalence of charge decreases. In the C_H_1 domains of bony fishes, there are still many charged residues (in C, CD, DE, E and EF), however the B strand and BC-loop reveal a lower number of charges and show instead an increase in hydrophobicity. No major charge changes can be found in the other domains of bony fishes. Although some point mutations may be present, there are no significant differences observable in tetrapods. 

As has already been said, the IgNAR is the one that differentiates the most between the different isotypes, exhibiting many charged residues. Although they are evolutionarily distant, the increase in charges is also significant in the C_H_3 domains of IgG and IgE, especially in the AB and DE-turn and in the C and E strand. On the other side, few charged residues are present in the G strand of IgG C_H_1 domains, but no other modification is noticeable. In fact, as literature confirms, while the IgG C_H_3–C_H_3 interface is mainly characterized by salt bridges, the C_H_1–C_L_ interface is mostly hydrophobic with the only salt bridge present between the G strand and the AB-turn [98,99]. The light chain isotypes are quite similar, with only few distinctions observed in the BC and DE strands. In these strands, Igκ has a more substantial proportion of charged residues. In contrast, in the G strand, Igλ exhibits a higher number of charged residues.

Some of the changes in biophysical properties between classes and/or isotypes can have a strong impact in the intra- and interdomain contacts. For example, residue 11 in strand A is a conserved tyrosine in the IgG C_H_3 domains and it makes van der Waals interactions with residues 16, 18 and 19 in the opposite domain (IMGT: 10, 12 and 13); in particular, 18 and 19 (IMGT: 12 and 13) are conserved negatively charged residues. In the IgNAR Y11 (IMGT: Y5) is substituted by a R, which can, if the interdomain orientation is preserved, make stronger salt bridges with the same partner residues (16,18 and 19, IMGT: 10, 12 and 13). Another example is the T60 (IMGT: T81) in the D strand of IgG that, in IgA and IgNAR, is substituted by a bulky F or W. This residue normally interacts with itself in the opposite domain making van der Waals interactions. The presence of a hydrophobic bulky residue in the same position allows a pi stacking interaction which may further stabilize the interdomain interface. Residue 79 (IMGT: 88) represents a case in which evolution inserted a highly favorable residue in order to obtain the strongest interactions. In fact, IgG and IgE, the most recent Ig isotypes, present in this position high occurrent positively charged residues (R or K), whereas other isotypes are mainly characterized by apolar residues (L) or low occurrent charges. Residue 79 (IMGT: 88) can make a salt bridge with the negatively charged residue in position 65 (IMGT: 84.2) and an additional pi stacking with the conserved F75 (IMGT: 85.1). Position 65 and 75 (IMGT: 84.2 and 85.1) already showed a negatively charged residue and the aromatic F in the other isotypes, but the negatively charged residue in position 79 (IMGT: 88) only became predominant in the more recent IgG and IgE. 

## 5. Conclusions

In conclusion, the study of biophysical properties across different isotypes and animal classes has provided valuable insights into the preserved and variable sites within the primary structure of immunoglobulins. 

Coevolving residue analysis has shown that, on the one hand, intradomain contacts are essential for proper Ig monomer folding and, on the other, interdomain interactions are crucial for the proper assembly and therefore functionality of the Ig molecule. The conservation score has revealed that the most conserved strands are those forming the structural core, and that solvent-pointing residue positions are more likely to undergo mutation. 

Our systematic analysis of biophysical properties has identified significant differences in sequences between ancient sharks and bony fishes compared to those of tetrapods, with the first ones presenting more charged residues that are instead less frequent in the higher vertebrates. Moreover, the analysis of other isotype sequences revealed mutations that contribute to strengthen the interface contacts. Overall, this research adds to the existing knowledge in the field of immunology and paves the way for future studies to learn from the first designer: evolution.

## Data Availability

All data is available in the manuscript or in the Supplementary Materials. Additional information is available upon request.

## Supplementary Materials

The following supporting information can be downloaded at: https://www.mdpi.com/article/10.3390/biom13050801/s1, Table S1. Number of sequences in the dataset; Table S2. Average conservation score; Figure S1. IMGT numeration; Figure S2. Evolutionary analysis of interdomain contacts; Figure S3. Numeration; Figure S4. PCA analysis of the CH3 domains sequences; Figure S5. Biophysical properties of CH3 domains in different isotypes; Figure S6. Biophysical properties of CH3 domains in different animal classes; Figure S7. PCA analysis of the CH1 domains sequences; Figure S8. Biophysical properties of CH1 domains in different isotypes; Figure S9. Biophysical properties of CH1 domains in different animal classes; Figure S10. PCA analysis of the CL domains sequences; Figure S11. Biophysical properties of CL domains in different isotypes; Figure S12. Biophysical properties of CL domains in different animal classes

## References

[1] Davies D.R., Chacko S. (1993). Antibody Structure. Acc. Chem. Res..

[2] Röthlisberger D., Honegger A., Plückthun A. (2005). Domain Interactions in the Fab Fragment: A Comparative Evaluation of the Single-Chain Fv and Fab Format Engineered with Variable Domains of Different Stability. J. Mol. Biol..

[3] Colman P.M., Dixon F.J. (1988). Structure of Antibody-Antigen Complexes: Implications for Immune Recognition. Advances in Immunology.

[4] Teplyakov A., Zhao Y., Malia T.J., Obmolova G., Gilliland G.L. (2013). IgG2 Fc Structure and the Dynamic Features of the IgG CH2–CH3 Interface. Mol. Immunol..

[5] Bruhns P., Iannascoli B., England P., Mancardi D.A., Fernandez N., Jorieux S., Daëron M. (2009). Specificity and Affinity of Human Fcγ Receptors and Their Polymorphic Variants for Human IgG Subclasses. Blood.

[6] Schroeder H.W., Cavacini L. (2010). Structure and Function of Immunoglobulins. J. Allergy Clin. Immunol..

[7] Alzari P.M., Delves P.J. (1998). Domains, Immunoglobulin-Type. Encyclopedia of Immunology.

[8] Bork P., Holm L., Sander C. (1994). The Immunoglobulin Fold: Structural Classification, Sequence Patterns and Common Core. J. Mol. Biol..

[9] Feige M.J., Hendershot L.M., Buchner J. (2010). How Antibodies Fold. Trends Biochem. Sci..

[10] Choi H.-J., Seok S.-H., Kim Y.-J., Seo M.-D., Kim Y.-S. (2015). Crystal Structures of Immunoglobulin Fc Heterodimers Reveal the Molecular Basis for Heterodimer Formation. Mol. Immunol..

[11] Charles A., Janeway J., Travers P., Walport M., Shlomchik M.J. (2001). Structural Variation in Immunoglobulin Constant Regions. Immunobiology: The Immune System in Health and Disease.

[12] Fillatreau S., Six A., Magadan S., Castro R., Sunyer J.O., Boudinot P. (2013). The Astonishing Diversity of Ig Classes and B Cell Repertoires in Teleost Fish. Front. Immunol..

[13] Justiz Vaillant A.A., Jamal Z., Patel P., Ramphul K. (2022). Immunoglobulin. StatPearls.

[14] Kumar Bharathkar S., Parker B.W., Malyutin A.G., Haloi N., Huey-Tubman K.E., Tajkhorshid E., Stadtmueller B.M. (2020). The Structures of Secretory and Dimeric Immunoglobulin A. eLife.

[15] Kumar N., Arthur C.P., Ciferri C., Matsumoto M.L. (2020). Structure of the Secretory Immunoglobulin A Core. Science.

[16] Li Y., Wang G., Li N., Wang Y., Zhu Q., Chu H., Wu W., Tan Y., Yu F., Su X.-D. (2020). Structural Insights into Immunoglobulin M. Science.

[17] Lyu M., Malyutin A., Stadtmueller B. (2023). The Structure of the Teleost Immunoglobulin M Core Provides Insights on Polymeric Antibody Evolution, Assembly, and Function. bioRxiv.

[18] Charles A., Janeway J., Travers P., Walport M., Shlomchik M.J. (2001). The Distribution and Functions of Immunoglobulin Isotypes. Immunobiology: The Immune System in Health and Disease.

[19] Pabst O. (2012). New Concepts in the Generation and Functions of IgA. Nat. Rev. Immunol..

[20] Mukai K., Tsai M., Starkl P., Marichal T., Galli S.J. (2016). IgE and Mast Cells in Host Defense against Parasites and Venoms. Semin. Immunopathol..

[21] Goding J.W. (1978). Allotypes of IgM and IgD Receptors in the Mouse: A Probe for Lymphocyte Differentiation. Contemp. Top. Immunobiol..

[22] Chen K., Cerutti A. (2011). The Function and Regulation of Immunoglobulin D. Curr. Opin. Immunol..

[23] Flajnik M.F., Kasahara M. (2010). Origin and Evolution of the Adaptive Immune System: Genetic Events and Selective Pressures. Nat. Rev. Genet..

[24] Amemiya C.T., Alföldi J., Lee A.P., Fan S., Philippe H., MacCallum I., Braasch I., Manousaki T., Schneider I., Rohner N. (2013). The African Coelacanth Genome Provides Insights into Tetrapod Evolution. Nature.

[25] Irisarri I., Baurain D., Brinkmann H., Delsuc F., Sire J.-Y., Kupfer A., Petersen J., Jarek M., Meyer A., Vences M. (2017). Phylotranscriptomic Consolidation of the Jawed Vertebrate Timetree. Nat. Ecol. Evol..

[26] Berstein R.M., Schluter S.F., Shen S., Marchalonis J.J. (1996). A New High Molecular Weight Immunoglobulin Class from the Carcharhine Shark: Implications for the Properties of the Primordial Immunoglobulin. Proc. Natl. Acad. Sci. USA.

[27] Matz H., Munir D., Logue J., Dooley H. (2021). The Immunoglobulins of Cartilaginous Fishes. Dev. Comp. Immunol..

[28] Ohta Y., Flajnik M. (2006). IgD, like IgM, Is a Primordial Immunoglobulin Class Perpetuated in Most Jawed Vertebrates. Proc. Natl. Acad. Sci. USA.

[29] Greenberg A.S., Avila D., Hughes M., Hughes A., McKinney E.C., Flajnik M.F. (1995). A New Antigen Receptor Gene Family That Undergoes Rearrangement and Extensive Somatic Diversification in Sharks. Nature.

[30] Roux K.H., Greenberg A.S., Greene L., Strelets L., Avila D., McKinney E.C., Flajnik M.F. (1998). Structural Analysis of the Nurse Shark (New) Antigen Receptor (NAR): Molecular Convergence of NAR and Unusual Mammalian Immunoglobulins. Proc. Natl. Acad. Sci. USA.

[31] Gambón Deza F., Sánchez Espinel C., Valdueza Beneitez J. (2007). A Novel IgA-like Immunoglobulin in the Reptile Eublepharis Macularius. Dev. Comp. Immunol..

[32] Belov K., Hellman L., Cooper D.W. (2002). Characterisation of Echidna IgM Provides Insights into the Time of Divergence of Extant Mammals. Dev. Comp. Immunol..

[33] Lundqvist M.L., Middleton D.L., Radford C., Warr G.W., Magor K.E. (2006). Immunoglobulins of the Non-Galliform Birds: Antibody Expression and Repertoire in the Duck. Dev. Comp. Immunol..

[34] Warr G.W., Magor K.E., Higgins D.A. (1995). IgY: Clues to the Origins of Modern Antibodies. Immunol. Today.

[35] Mußmann R., Du Pasquier L., Hsu E. (1996). Is Xenopus IgX an Analog of IgA?. Eur. J. Immunol..

[36] Das S., Hirano M., Tako R., McCallister C., Nikolaidis N. (2012). Evolutionary Genomics of Immunoglobulin-Encoding Loci in Vertebrates. Curr. Genom..

[37] Mirete-Bachiller S., Olivieri D.N., Gambón-Deza F. (2021). Immunoglobulin T Genes in Actinopterygii. Fish Shellfish. Immunol..

[38] Tongsri P., Meng K., Liu X., Wu Z., Yin G., Wang Q., Liu M., Xu Z. (2020). The Predominant Role of Mucosal Immunoglobulin IgT in the Gills of Rainbow Trout (Oncorhynchus Mykiss) after Infection with Flavobacterium Columnare. Fish Shellfish. Immunol..

[39] Oreste U., Ametrano A., Coscia M.R. (2021). On Origin and Evolution of the Antibody Molecule. Biology.

[40] Marks D.S., Colwell L.J., Sheridan R., Hopf T.A., Pagnani A., Zecchina R., Sander C. (2011). Protein 3D Structure Computed from Evolutionary Sequence Variation. PLoS ONE.

[41] Balakrishnan S., Kamisetty H., Carbonell J.G., Lee S.-I., Langmead C.J. (2011). Learning Generative Models for Protein Fold Families. Proteins: Struct. Funct. Bioinform..

[42] Morcos F., Pagnani A., Lunt B., Bertolino A., Marks D.S., Sander C., Zecchina R., Onuchic J.N., Hwa T., Weigt M. (2011). Direct-Coupling Analysis of Residue Coevolution Captures Native Contacts across Many Protein Families. Proc. Natl. Acad. Sci. USA.

[43] Hopf T.A., Colwell L.J., Sheridan R., Rost B., Sander C., Marks D.S. (2012). Three-Dimensional Structures of Membrane Proteins from Genomic Sequencing. Cell.

[44] Ovchinnikov S., Kamisetty H., Baker D. (2014). Robust and Accurate Prediction of Residue–Residue Interactions across Protein Interfaces Using Evolutionary Information. eLife.

[45] Ovchinnikov S., Kinch L., Park H., Liao Y., Pei J., Kim D.E., Kamisetty H., Grishin N.V., Baker D. (2015). Large-Scale Determination of Previously Unsolved Protein Structures Using Evolutionary Information. eLife.

[46] Hopf T.A., Ingraham J.B., Poelwijk F.J., Schärfe C.P.I., Springer M., Sander C., Marks D.S. (2017). Mutation Effects Predicted from Sequence Co-Variation. Nat. Biotechnol..

[47] Johnson M., Zaretskaya I., Raytselis Y., Merezhuk Y., McGinnis S., Madden T.L. (2008). NCBI BLAST: A Better Web Interface. Nucleic Acids Res..

[48] Sievers F., Wilm A., Dineen D., Gibson T.J., Karplus K., Li W., Lopez R., McWilliam H., Remmert M., Söding J. (2011). Fast, Scalable Generation of High-Quality Protein Multiple Sequence Alignments Using Clustal Omega. Mol. Syst. Biol..

[49] Okonechnikov K., Golosova O., Fursov M., the UGENE Team (2012). Unipro UGENE: A Unified Bioinformatics Toolkit. Bioinformatics.

[50] Sievers F., Higgins D.G. (2018). Clustal Omega for Making Accurate Alignments of Many Protein Sequences. Protein Sci..

[51] Capella-Gutiérrez S., Silla-Martínez J.M., Gabaldón T. (2009). TrimAl: A Tool for Automated Alignment Trimming in Large-Scale Phylogenetic Analyses. Bioinformatics.

[52] The UniProt Consortium (2023). UniProt: The Universal Protein Knowledgebase in 2023. Nucleic Acids Res..

[53] Lefranc M.-P., Pommié C., Kaas Q., Duprat E., Bosc N., Guiraudou D., Jean C., Ruiz M., Da Piédade I., Rouard M. (2005). IMGT Unique Numbering for Immunoglobulin and T Cell Receptor Constant Domains and Ig Superfamily C-like Domains. Dev. Comp. Immunol..

[54] Lefranc M.-P. (2014). Immunoglobulin and T Cell Receptor Genes: IMGT^®^ and the Birth and Rise of Immunoinformatics. Front. Immunol..

[55] Lefranc M.-P., Giudicelli V., Ginestoux C., Jabado-Michaloud J., Folch G., Bellahcene F., Wu Y., Gemrot E., Brochet X., Lane J. (2009). IMGT^®^, the International ImMunoGeneTics Information System^®^. Nucleic Acids Res..

[56] Lefranc M.-P. (2008). IMGT^®^, the International ImMunoGeneTics Information System^®^ for Immunoinformatics. Mol. Biotechnol..

[57] Capra J.A., Singh M. (2007). Predicting Functionally Important Residues from Sequence Conservation. Bioinformatics.

[58] Minh B.Q., Schmidt H.A., Chernomor O., Schrempf D., Woodhams M.D., von Haeseler A., Lanfear R. (2020). IQ-TREE 2: New Models and Efficient Methods for Phylogenetic Inference in the Genomic Era. Mol. Biol. Evol..

[59] Truszkowski J., Goldman N. (2016). Maximum Likelihood Phylogenetic Inference Is Consistent on Multiple Sequence Alignments, with or without Gaps. Syst. Biol..

[60] Kumar S., Stecher G., Li M., Knyaz C., Tamura K. (2018). MEGA X: Molecular Evolutionary Genetics Analysis across Computing Platforms. Mol. Biol. Evol..

[61] Whelan S., Goldman N. (2001). A General Empirical Model of Protein Evolution Derived from Multiple Protein Families Using a Maximum-Likelihood Approach. Mol. Biol. Evol..

[62] Schwarz G. (1978). Estimating the Dimension of a Model. Ann. Stat..

[63] Soubrier J., Steel M., Lee M.S.Y., Der Sarkissian C., Guindon S., Ho S.Y.W., Cooper A. (2012). The Influence of Rate Heterogeneity among Sites on the Time Dependence of Molecular Rates. Mol. Biol. Evol..

[64] Minh B.Q., Nguyen M.A.T., von Haeseler A. (2013). Ultrafast Approximation for Phylogenetic Bootstrap. Mol. Biol. Evol..

[65] Hoang D.T., Chernomor O., von Haeseler A., Minh B.Q., Vinh L.S. (2018). UFBoot2: Improving the Ultrafast Bootstrap Approximation. Mol. Biol. Evol..

[66] Matsumiya S., Yamaguchi Y., Saito J., Nagano M., Sasakawa H., Otaki S., Satoh M., Shitara K., Kato K. (2011). Corrigendum to “Structural Comparison of Fucosylated and Nonfucosylated Fc Fragments of Human Immunoglobulin G1” [J. Mol. Biol. 386/3 (2007) 767–779]. J. Mol. Biol..

[67] Suzek B.E., Wang Y., Huang H., McGarvey P.B., Wu C.H. (2015). UniRef Clusters: A Comprehensive and Scalable Alternative for Improving Sequence Similarity Searches. Bioinformatics.

[68] Ekeberg M., Lövkvist C., Lan Y., Weigt M., Aurell E. (2013). Improved Contact Prediction in Proteins: Using Pseudolikelihoods to Infer Potts Models. Phys. Rev. E.

[69] Hopf T.A., Green A.G., Schubert B., Mersmann S., Schärfe C.P.I., Ingraham J.B., Toth-Petroczy A., Brock K., Riesselman A.J., Palmedo P. (2019). The EVcouplings Python Framework for Coevolutionary Sequence Analysis. Bioinformatics.

[70] GetContacts. https://getcontacts.github.io/.

[71] Tareen A., Kinney J.B. (2020). Logomaker: Beautiful Sequence Logos in Python. Bioinformatics.

[72] Python 3 Reference Manual:|Guide Books. https://dl.acm.org/doi/book/10.5555/1593511.

[73] Pedregosa F., Varoquaux G., Gramfort A., Michel V., Thirion B., Grisel O., Blondel M., Prettenhofer P., Weiss R., Dubourg V. (2011). Scikit-Learn: Machine Learning in Python. J. Mach. Learn. Res..

[74] Wimley W.C., White S.H. (1996). Experimentally Determined Hydrophobicity Scale for Proteins at Membrane Interfaces. Nat. Struct. Mol. Biol..

[75] Fauchère J.L., Charton M., Kier L.B., Verloop A., Pliska V. (1988). Amino Acid Side Chain Parameters for Correlation Studies in Biology and Pharmacology. Int. J. Pept. Protein Res..

[76] Cooper L.J., Shikhman A.R., Glass D.D., Kangisser D., Cunningham M.W., Greenspan N.S. (1993). Role of Heavy Chain Constant Domains in Antibody-Antigen Interaction. Apparent Specificity Differences among Streptococcal IgG Antibodies Expressing Identical Variable Domains. J. Immunol..

[77] Brinkmann U., Kontermann R.E. (2017). The Making of Bispecific Antibodies. MAbs.

[78] Zhang X., Calvert R.A., Sutton B.J., Doré K.A. (2017). IgY: A Key Isotype in Antibody Evolution. Biol. Rev..

[79] Keyt B.A., Baliga R., Sinclair A.M., Carroll S.F., Peterson M.S. (2020). Structure, Function, and Therapeutic Use of IgM Antibodies. Antibodies.

[80] Scapin G., Yang X., Prosise W.W., McCoy M., Reichert P., Johnston J.M., Kashi R.S., Strickland C. (2015). Structure of Full-Length Human Anti-PD1 Therapeutic IgG4 Antibody Pembrolizumab. Nat. Struct. Mol. Biol..

[81] Criscitiello M.F., Flajnik M.F. (2007). Four Primordial Immunoglobulin Light Chain Isotypes, Including λ and κ, Identified in the Most Primitive Living Jawed Vertebrates. Eur. J. Immunol..

[82] Stavnezer J., Amemiya C.T. (2004). Evolution of Isotype Switching. Semin. Immunol..

[83] Thomas J., Ramakrishnan N., Bailey-Kellogg C. (2008). Graphical Models of Residue Coupling in Protein Families. IEEE/ACM Trans. Comput. Biol. Bioinform..

[84] Wilkins A., Erdin S., Lua R., Lichtarge O. (2012). Evolutionary Trace for Prediction and Redesign of Protein Functional Sites. Methods Mol. Biol..

[85] Göbel U., Sander C., Schneider R., Valencia A. (1994). Correlated Mutations and Residue Contacts in Proteins. Proteins.

[86] Hopf T.A., Schärfe C.P.I., Rodrigues J.P.G.L.M., Green A.G., Kohlbacher O., Sander C., Bonvin A.M.J.J., Marks D.S. (2014). Sequence Co-Evolution Gives 3D Contacts and Structures of Protein Complexes. eLife.

[87] Marks D.S., Hopf T.A., Sander C. (2012). Protein Structure Prediction from Sequence Variation. Nat. Biotechnol..

[88] Jumper J., Evans R., Pritzel A., Green T., Figurnov M., Ronneberger O., Tunyasuvunakool K., Bates R., Žídek A., Potapenko A. (2021). Highly Accurate Protein Structure Prediction with AlphaFold. Nature.

[89] Merchant A.M., Zhu Z., Yuan J.Q., Goddard A., Adams C.W., Presta L.G., Carter P. (1998). An Efficient Route to Human Bispecific IgG. Nat. Biotechnol..

[90] Ma J., Mo Y., Tang M., Shen J., Qi Y., Zhao W., Huang Y., Xu Y., Qian C. (2021). Bispecific Antibodies: From Research to Clinical Application. Front. Immunol..

[91] Bertz M., Buchner J., Rief M. (2013). Mechanical Stability of the Antibody Domain CH3 Homodimer in Different Oxidation States. J. Am. Chem. Soc..

[92] Rispens T., Davies A.M., Ooijevaar-de Heer P., Absalah S., Bende O., Sutton B.J., Vidarsson G., Aalberse R.C. (2014). Dynamics of Inter-Heavy Chain Interactions in Human Immunoglobulin G (IgG) Subclasses Studied by Kinetic Fab Arm Exchange. J. Biol. Chem..

[93] Anishchenko I., Ovchinnikov S., Kamisetty H., Baker D. (2017). Origins of Coevolution between Residues Distant in Protein 3D Structures. Proc. Natl. Acad. Sci. USA.

[94] Lesk A.M., Chothia C. (1982). Evolution of Proteins Formed by β-Sheets: II. The Core of the Immunoglobulin Domains. J. Mol. Biol..

[95] Thies M.J.W., Talamo F., Mayer M., Bell S., Ruoppolo M., Marino G., Buchner J. (2002). Folding and Oxidation of the Antibody Domain CH3. J. Mol. Biol..

[96] Feige M.J., Groscurth S., Marcinowski M., Shimizu Y., Kessler H., Hendershot L.M., Buchner J. (2009). An Unfolded CH1 Domain Controls the Assembly and Secretion of IgG Antibodies. Mol. Cell.

[97] Feige M.J., Gräwert M.A., Marcinowski M., Hennig J., Behnke J., Ausländer D., Herold E.M., Peschek J., Castro C.D., Flajnik M. (2014). The Structural Analysis of Shark IgNAR Antibodies Reveals Evolutionary Principles of Immunoglobulins. Proc. Natl. Acad. Sci. USA.

[98] Fernández-Quintero M.L., Quoika P.K., Wedl F.S., Seidler C.A., Kroell K.B., Loeffler J.R., Pomarici N.D., Hoerschinger V.J., Bujotzek A., Georges G. (2022). Comparing Antibody Interfaces to Inform Rational Design of New Antibody Formats. Front. Mol. Biosci..

[99] Pomarici N.D., Waibl F., Quoika P.K., Bujotzek A., Georges G., Fernández-Quintero M.L., Liedl K.R. (2023). Structural Mechanism of Fab Domain Dissociation as a Measure of Interface Stability. J. Comput. Aided Mol. Des..

