# Supplementary material for: Evolution of the Immunoglobulin Isotypes—Variations of Biophysical Properties among Animal Classes
Source: Biomolecules. 2023 May 8;13(5):801. doi: 10.3390/biom13050801 (PMC10216798; doi:10.3390/biom13050801)
Supplement: Supplementary file 1 [file biomolecules-13-00801-s001.zip › biomolecules-2357674-supplementary.pdf]

Evolution of the Immunoglobulin Isotypes –

Variations of Biophysical Properties between Animal Classes

Nancy D. Pomarici[1], Roberta Cacciato[1], Janik Kokot[1], Monica L. Fernández-Quintero[1,*] and Klaus R. Liedl[1,]

[1]Institute of General, Inorganic and Theoretical Chemistry, Center for Molecular Biosciences Innsbruck (CMBI), University of Innsbruck, Innrain 80-82, A-6020 Innsbruck, Austria.

*Correspondence: Monica.Fernandez-Quintero@uibk.ac.at, Klaus.Liedl@uibk.ac.at

C_H1 domains accession codes:

CAC43066; NWZ28413; KFQ46330; NWI29386; KAI6061223; NXD18504; QAT77293; AAA68606; NXA57377; AEF79983; AMS75109; ASU87373; APB61243; AFZ39218; AAB22614; AFZ39174; AFZ39211; AFA41928; AFZ39189; AFZ39165; AFZ39193; AFZ39176; AFA41929; KAF7235451; ABG72683; CAC20453; P01876; PNI15543; AAB30803; AAB59396; CAA37745; CAA37741; CAA37744; P20758; CAA37743; CAA37746; AAQ62620; ABI97137; ABI97140; ABI97138; ABI97139; QJB76121; QJB76122; CAA37742; ABK15554; ABI97146; ABI97149; AAK72414; ABI97145; AAK72413; AAK72412; AAQ62621; AAT11507; AAK72410; ABI97144; ATV90895; ABI97143; ABI97142; ABI97141; AAK72411; QJB76118; AAC64980; AAT65195; AAC98391; ADD51207; AAA65943; AMP34155; ELR44852; NIG60332; ATI97333; ATI97345; ATI97346; ATI97338; ATI97337; AAA56796; KAI5162510; AAX73304; QJY40729; CAO79580; ADD71719; ELK01275; KAF6072915; AAP80145; P0DUB3; EPQ16373; AQR55605; AQR55606; BAL70397; AQR55608; AQR55609; ALB75436; ELK36012; EHB17602; ELV12711; QGV56640; EHB08048; KFO35296; AGN11433; KAG8525088; AAL15540; AAL15539; AAD41690; AAB84185; AAL17701; AAN33012; AAL17700; AAC48835; AAD21190; BAD34542; BAD34541; ACO88906; AFQ38975; AFI33218; AXL14358; UZS59180; AHY86392; AFZ39186; AFZ39208; APB61242; AKM28416; AFR33844; ABY67439; BAV35204; BAV35207; ABC75541; AIW06017; AAB21246; PNI88328; PNJ04970; ABB89457; ABB89464; ABB89458; ABB89461; ATV90901; XP_032123471; ABB89467; AAN07167; AAN03672; QJY40730; AAU09793; XP_043413080; AAX73311; AAN07165; XP_044789334; AMP34156; AAN03671; ANN46372; AAN03673; AAB59653; AAO19643; AES92971; AES92974; AES92977; AES92976; AES92973; AES92972; AGY29606; AGG53192; AGY29601; AGY29615; AGY29616; AGY29605; AGG53194; AGG53198; AGG53201; AGG53184; AGG53222; AGG53231; AGG53219; AGG53227; AGG53223; AGG53202; AGG53218; AGG53226; AGG53229; AGG53224; AGG53195; AGG53185; AGY29603; AAB03680; BAJ20182; BAJ20183; AGG53193; AGG53204; AGG53186; AGG53199; AGG53206; AGY29600; AES92963; AES92968; AES92981; AGG53211; AGG53212; AGG53207; AGG53208; AGG53213; AGG53217; AES92962; AES92979; AGG53203; AES92982; AAT02197; AES92983; AES92970; AES92967; ACU45375; AFR90288; AFR90269; AFR90270; AFR90264; AFR90268; AFR90274; AFR90279; AFR90257; AFR90258; AAB59395; P01854; P0DOX4; AEG78107; AAA35416; ATV90896; AEG78114; AEG78120; AEG78096; AEG78100; AEG78105; AEG78110; AEG78095; EGW12809; AEG78093; AEG78099; CAC44499; AEG78094; AAA85662; AAF43901; AAA53301; AEG78097; QGU21335; QGU21326; QGU21344; QGU21334; QGU21345; QGU21347; QGU21352; AEG78098; QGU21351; QGU21350; QGU21342; QGU21318; QGU21322; QGU21317; QGU21325; QGU21333; AEH26435; KAF0884900; AAA41364; P01855; QGU21320; QGU21321; AAX73306; QGU21280; QGU21289; QGU21348; QGU21309; CAO79579; AAA56797; QGU21307; QGU21302; QGU21287; QGU21349; ELK01276; AEG78103; AEG78101; KAG8525035; ELK37506; ALB75437; KAH0514093; QGV56638; KAF6500411; CAA81788; KAI5936975; ELV12710; P06336; BAQ55489; KAI5162512; QJY40731; KAF6273895; KAF6279810; BAM66311; KAF6268940; AAO37095; AAB09546; AAL17702; ELR44851; AAA51378; AAM45140; AAC79674; EPQ01894; PNI15546; AXN93645; AXN93652; AAG00910; PNJ01622; 5DK3; AXN93654; PNJ04973; AAF14058; PNI15539; AAT11504; AAM93486; AAT11502; AAM93487; PNI15541; CAC20457; AIL50394; AAT11503; AAM93488; AAM93489; AAQ57562; AGC00826; AAT11505; AAF14060; AAG00912; AIK19747; AGC00825; AGC00822; ATV90899; ACO54886; AGC00821; AAQ57556; CAC20456; QNM80716; AGC00823; QJB76128; ATV90897; AGC00827; AIM44673; AIM44672; MBZ3888618; AAL35302; EHB08049; AAS18414; AIM44676; AKM12424; AAB59265; AKM12428; AIM44631; ABD64612; AYM94157; AAL35303; ADD71697; BAA32230; CAC44763; XP_042845222; AIM44657; AIM44666; AIM44652; BAI82568; BAM75541; BAM75540; AIM44639; AAX73307; AAA52216; BAM75546; BAM75565; ABY85807; ATI97579; AHH34165; AHA35861; AIM44674; AIM44675; AIM44644; AIM44661; AIM44662; AAA52219; P01862; AAG42243; BAM66310; ALB75439; ADX94416; AAA30965; ABY85805; ATI97593; AQR55684; ACD64981; AAX73308; AAA51294; P20759; P20760; ABD64610; ADX94417; KAB1277999; AIM44671; EHB08050; ALB75438; KFO35298; NIG61489; EPQ16374; CAC44760; CAC44624; AAP82181; AAL35301; AAL35304; EGW12805; BAM66309;

AAA51295; AAA82733; AAG01011; AAB37381; EPQ01893; CAO79572; ABE68619; AQT27060; EPY72686; AQT27058; CAC86339; CAC86340; KAH0514073; ANN46376; P20762; ADZ32495; ACO52352; AAB59697; BAA03476; AAT65197; AAC48761; CAA49451; BAM66305; EGW12808; AAC48762; AAB59660; ANN46375; AAT65196; ABY85808; AAB37380; ANN46379; AQT27056; BAI82569; AWY10546; ABD73925; AAA37906; AAA60738; AQT27057; CAC86341; EGW12806; CAA34864; QGV56636; AAB59658; ABQ85912; CAA47649; KAG8519298; AAB59656; P01868; BAL70394; ADX94418; KAI5162393; AAL17703; AAL17704; AAD55590; ACX54357; AAM61760; QWC92995; AFR90251; AFR90252; AFR90253; AFR90246; ACU45374; AFR90247; AFR90248; AFR90245; AFR33767; AFR33765; ABV66132; AFR33766; ABV66129; AIW06024; AIW06020; AIW06021; AIW06022; CAE02686; BAV35203; CAA49247; AFZ39171; AFZ39181; AFA41930; AFZ39220; AFZ39182; KAI6061222; CAA46322; APB61246; APB61247; AMS75110; AHZ12733; AKM28417; QAT77294; AFZ39169; AFZ39170; AFZ39180; AFZ39224; KYO29711; BDI00299; ABW77756; ABY54906; AAC59687; XP_039855852; ABD76396; AEH76773; ABW89619; ABW81216; ACA51888; XP_037834750; ACM90147; AAL99929; ABW81218; XP_026021082; ABW89622; ANO53445; XP_037544448; ABW81220; AAL99934; AHC31440; XP_025762367; XP_025762368; AHY86391; XP_042565114; AAC59688; MBN3322266; RXN05506; AHI18415; TSK87411; QHO62363; BAB60868; BCU66532; ARC77253; QDP16243; APJ36370; KAF5906456; AOW44096; AOW44093; AOW44094; UUY86300; KAA0705619; XP_029913635; XP_029913634; XP_029913633; AFA41926; QAT77292; AMK48542; APB61245; AMS75108; NXW26075; APB61244; NWS64666; CAC43281; ASU87372; ASU87371; KAI6061224; AFZ39177; AFZ39178; AFZ39179; AFZ39216; ABV66128; ABY74509; AFR33764; AFR33841; KAG8140957; XP_017945116; BAV32322; CAE02685; AAA49878; AIW06014; AFM37312; CAC20458; PNJ04967; PNI10622; QJB76129; AAD02420; KAH0514101; EGW12804; AAB59651; CAA27326; QGU21427; QGU21428; QGU21429; QGU21431; QGU21430; QGU21377; QGU21356; QGU21366; QGU21374; QGU21364; QGU21382; QGU21358; QGU21397; QGU21360; QGU21353; QGU21355; QGU21359; QGU21375; AAU09792; QGU21407; QGU21398; QGU21426; QGU21420; QGU21413; QGU21404; QGU21405; QGU21403; QGU21402; QGU21432; KAI5934885; KAI5936976; KAI5162392; BAL70395; P06337; AAG40853; EPQ16375; MBZ3882660; KAI5936977; BAA32231; ANN46370; AAB09545; AAX73309; QJY40733; KAG8519297; AAA51379; CAO79569; AAO37096; EHB10927; AAB62251; ADD71716; ABX89978; ALB75443; CAA42611; EHB11820; QGV56635; KFO35302; BAD00196; AIA77883; AAF80358; BAM66301; AAC48775; QWC92960; QWC92964; AAD21191; AAO37747; AAD24482; P20768; AAN33013; XP_041031595; AGG53273; ABW84256; AES92946; ABW84248; AGG53275; AGG53277; AGG53281; ABW84251; AAU04508; ABW84249; AGG53282; AGG53286; AGG53285; ABW84252; AGG53279; ABW84254; AES92940; AGG53276; AGG53278; AES92935; AES92956; UTD43067; AES92941; AES92937; ABW84253; BAJ20181; AES92934; AES92945; AES92955; AES92957; AES92939; AES92951; AES92944; AES92947; AES92936; AES92950; AES92954; AES92943; AEJ86609; AEJ86613; ABW84255; AAK07642; ABW84250; AES92952; AAB04673; AEJ86611; AEJ86616; AEJ86614; AEJ86607; AEJ86608; AEJ86600; ACU11614; AEJ86610; ACU11613; ACU11612; AEJ86605; AEJ86601; AEJ86599; AEJ86615; AEJ86629; AEJ86606; ACU11611; AFR90255; EMP41995; AAB03838; ACU45376; 2RCJ; AAB48195; ABB83616; AYN64868; AYN64869; AES92988; AES92986; AES92959; AES92984; AES92989; AES92987; UTD43068; BAJ20187; AGG53233; AGG53246; AGG53238; AGG53232; AGG53260; AGG53264; AGG53263; AGG53261; AAT02204.

CH3 domains accession codes:

NWZ28413; KFQ46330; CAC43065; NWI29386; KAI6061223; NXD18504; QAT77293; NXA57377; AEF79983; AMS75109; ASU87373; OWK49842; APB61243; AFZ39218; AAB22614; AFZ39174; AFA41928; CAC43064; AFZ39189; EOA93117; AFZ39165; AFZ39193; ACE79178; AFZ39176; AFZ39219; AFZ39210; AFZ39190; AFA41929; 6UE7; CAA37745; CAA37741; CAA37744; CAA37746; 6UE8; AAQ62620; QJB76121; ABI97146; QJB76120; ABI97143; AAK72411; ATV91193; AAC64980; AAT65195; AAC98391; BAD67216; MBW04533; AMP34155; ELR44852; NIG60332; ATI97333;

AAA56796; KAI5162510; ATI97347; KAF0884899; KAI5936974; KAB1277053; AAX73304; QJY40729; CAO79580; ADD71719; ELK01275; KAF6072915; AAP80145; P0DUB3; EPQ16373; AQR55605; BAL70397; AQR55608; AQR55607; ALB75436; ELK36012; ELV12711; EHB08048; KFO35296; AGN11433; AAA41375; AXM43016; ELK37507; P01879; AXM43009; AAL15542; AAL15540; AXM43013; AXM43008; AXM43012; AXM43015; AXM43014; ABV01995; AAD41690; AAB84185; AAL17701; AAN33012; AAL17700; AAC48835; AAD21190; QWC92952; QWC92951; BAD34542; ACO88906; AFQ38975; AFI33218; AXL14358; UZS59180; AAL79934; AHY86392; KAG7506059; AFR33844; ABY67439; AIW06017; AIW06018; AAB21246; P01880; PNI88328; PNJ04970; ABB89457; ABB89461; ATV90901; XP_032123471; ABB89467; AAN07167; QJY40730; AAU09793; XP_043413080; AAX73311; KFO35301; AAN07165; XP_044789334; AMP34156; AAN03670; AAN03671; AAB59653; AAO19643; AES92971; AES92974; AES92977; AES92975; AES92976; AES92973; AES92972; AGY29606; AES92978; AGY29601; AGY29615; AGY29605; AGG53222; AGG53223; AGG53228; AGG53230; AGY29603; AAB03680; BAJ20182; BAJ20184; ACU45375; AFR90280; AFR90266; AFR90268; AFR90291; AFR90290; 4GRG; 5NQW; 5LGK; P0DOX4; AEG78107; AEG78109; AAA35416; ATV90896; AEG78114; ATV91162; AEG78112; AEG78120; AEG78096; AEG78105; AEG78110; AEG78095; AEG78116; AEG78117; AEG78118; AEG78093; AEG78099; BAL70396; CAC44499; AEG78094; AAA85662; AAF43901; AAA53301; AEG78097; QGU21335; QGU21345; QGU21347; QGU21352; AEG78098; QGU21351; QGU21350; QGU21342; QGU21322; QGU21324; AEG78104; QGU21346; KAF0884900; BAB40315; QGU21320; AAX73306; QGU21280; CAO79579; AAA56797; QGU21279; QGU21307; QGU21349; QGU21291; ELK01276; CAO79578; AEG78103; KAG8525035; KAB1277055; ELK37506; KAH0514093; QGV56638; KAF6500411; CAA81788; AAA37916; AAA37912; KAI5936975; ELV12710; AAZ05128; BAQ55489; KAI5162512; QJY40731; KAF6273895; KAF6279810; BAM66311; EHB16230; KAF6268940; KFO35297; AAO37095; AAD27886; ANN46381; AAL17702; ELR44851; AAA51378; AAM45140; AAC79674; AAC78140; ABV01996; 1SY6; 5HSF; AAD38158; PNI15546; AXN93645; AAB86467; PNI15544; CAA43606; QNT08899; 7RHO; 5U4Y; 4BM7; 4DZ8; 7Q15; 4DZ8; AAG00910; AAB21082; PNJ01622; 7LUS; 5DK3; AXN93654; PNJ04973; AXN93657; AAF14058; PNJ04971; PNI15539; 7LUS; AAT11504; AAM93486; 4B53; PNI15541; CAC20457; BCY27132; AAA18103; AIL50394; AGC00824; AAT11503; AAM93488; AAM93489; AAQ57562; AGC00826; 6D58; 5W38; 6D4I; AXN93656; AAF14060; AAG00912; AIK19748; AIK19749; AIL50396; AIL50395; AIL50393; AGC00825; AAQ57565; AGC00822; QEV83695; CAA67886; QJB76126; AIK19746; AIL50397; AGC00821; AAQ57556; QJB76127; CAC20456; AAF14059; 5HVW; QNM80716; 6WMH; AGC00823; QJB76128; AAQ57570; AGC00827; 6WOL; 5K65; MBZ3888618; AAS18414; AIM44676; AAA31288; XP_051697838; AKM12436; AAA64252; BAA32230; CAC44763; CAC44762; XP_042845222; AIM44657; AIM44668; AIM44652; AIM44637; AIM44670; BAI82568; CAO79576; CAO79577; AIM44650; AIM44669; AIM44639; AIM44641; AIM44638; AIM44651; AIM44659; AAX73307; ABY85810; AAA52216; BAM75546; BAM75560; ABY85807; ATI97578; ATI97579; AHA35861; AIM44674; AIM44675; AIM44658; AIM44644; AIM44660; AIM44661; AIM44662; ABW89018; AAA52219; P01862; AAG42243; BAM66310; ALB75439; ABY85806; ADX94416; AAA30965; AAA51281; ABY85805; ATI97593; KAF0871663; AQR55684; AAX73260; CAO79571; BAE20056; AAA52220; AAX73308; AAA51294; CAO79570; AAA41373; AAA41376; BAM75571; ATI97556; KAB1277999; AIM44671; KAB1277054; AAX73259; EHB08050; ALB75442; ADX94415; KFO35298; NIG61489; EPQ16374; CCF72130; ALB75440; CAC44760; ALB75441; AAP82181; AAL35301; AAL35304; EGW12805; AAY98508; BAM66309; AAA82733; AAG01011; ELK30486; AAB37381; EPQ01893; CAO79572; ABE68619; EPY72686; CAC86339; CAC86340; KAH0514073; ANN46376; CAO79573; ADK11691; ADI87159; KFO35299; QJY40732; P20762; ACO52352; ADZ32494; AAT65197; ELK24149; AAC48761; CAA49451; BAM66305; EGW12808; AAC48762; AAB59660; ANN46375; P01865; KAB1278001; ACS34824; ANN46374; AAT65196; ABY85808; AAB37380; ANN46379; AQT27056; BAI82569; ANN46378; ACO52350; ABD73925; AAA41374; AAA37906; AAA37907; AQT27057; CAC86341; CAA24176; AAD40243; EGW12806; CAA34864; QGV56636; ACO52351; ACO52349; AAH92269; KAG8519298; KAB1252581; P01867; CAA39804; AAB59657; BAX57196; QGV56637; BAL70394; AAC36504; ADX94418; KAI5162393; KAI5162394; ELR44850; KAI5162514; AAL17703; AAL17704; AAD55590;

ABV01997; ACX54357; AAM61760; QWC92995; AFR90251; EMP30595; AFR90250; AFR90246; ACU45374; EMP30114; AFR90247; EMP40110; ALU11324; AFR33767; AFR33765; ABV66132; AFR33766; ABV66131; AIW06027; AIW06026; AIW06024; AIW06023; CAE02686; BAV35203; CAA49247; AFZ39171; AFZ39181; AFZ39200; AFZ39223; AFA41930; AFZ39220; KAI6061222; AFZ39221; CAA55148; APB61246; APB61247; NXK71518; AMS75110; AHZ12733; AKM28417; QAT77294; AFZ39169; AFZ39170; NXP02446; AFZ39180; AFZ39224; KYO29711; BDI00299; AEJ86627; ABW77756; ABY54906; AAC59687; XP_039855852; ABD76396; AEH76772; AEH76773; ABW89619; AEJ86624; ABI18250; ACA51888; AEH76774; XP_037834750; ACM90147; AAL99929; ABW77754; XP_026021082; ABW89622; ANO53445; XP_037544448; ABW81220; AHC31440; XP_025762367; XP_025762369; AHY86391; XP_042565114; AAW71781; AAC59688; MBN3322266; RXN05506; TSK87411; QHO62363; BAB60868; BCU66532; ARC77253; QDP16243; APJ36370; KAF5906456; AOW44096; AOW44093; AOW44094; UUY86300; KAA0705619; ATG71407; XP_029913635; AFA41926; AFA41927; QAT77292; CAC43068; CAC43062; EOA92985; AMK48542; APB61245; AMS75108; NXW26075; APB61244; NWS64666; CAC43281; ASU87372; AAA68605; ASU87371; KAI6061224; KQK84655; CAC43061; AMS75107; AFZ39177; AFZ39206; AFZ39178; AFZ39212; AFZ39179; AFZ39217; AFZ39213; ABV66128; KAF7235453; ABY74509; KAG8140955; AFR33764; AFR33841; KAG8140958; KAG8140957; XP_017945116; BAV32322; CAE02685; CAL92494; BAV32323; AAA49878; AIW06014; AIW06015; 7QDO; 7K0C; PNJ04967; PNJ04968; PNI10622; QJB76129; ATV91186; AAB59421; ATV91189; AAB59422; KAH0514101; EGW12804; CAA48392; 8E4C; 4JVW; CAC20701; AAA37908; AAA31293; MBW04751; QGU21427; QGU21428; QGU21429; P04221; QGU21367; AAU09792; QGU21407; QGU21432; QGU21363; QGU21416; QGU21406; ABQ42870; KAI5934885; BAL70395; P06337; AAG40853; EPQ16375; MBZ3882660; KAF6273897; EPQ01892; KAI5936977; KAF6273896; BAA32231; ANN46370; AAX73309; QJY40733; ANN46368; BAD00197; KAF6072922; KAG8519297; AAA51379; BAD00199; CAO79569; AAO37096; EHB10927; AAB62251; ANN46369; KAF6072921; ADD71716; ABX89978; BAD00198; CAA42611; EHB11820; KFO35300; QGV56635; KFO35302; QGV56641; BAD00196; AIA77883; ACS29496; ABV01998; AAF80358; BAE20181; AAA51297; QWC92960; AAD21191; AAO37747; AAD24482; AAC48834; CAA32113; AAN33013; ELK18451; XP_041031595; AGG53273; AGG53270; ABW84256; AGG53267; AES92946; ABW84248; AGG53275; ABW84251; ABW84249; AGG53286; AGG53279; AES92940; AGG53276; AGG53278; AGG53283; AES92935; AES92956; UTD43067; AES92941; AES92937; ABW84253; BAJ20181; AES92934; AES92945; AES92955; AES92957; AES92942; AES92944; AES92947; AES92950; AES92954; AES92943; AEJ86609; AEJ86613; ABW84255; AAK07642; ABW84250; AES92949; BAJ20180; AES92952; AES92933; AES92953; AEJ86612; AAB04673; AAB04672; AEJ86611; AEJ86616; AEJ86614; AEJ86607; AEJ86608; AEJ86600; ACU11614; AEJ86610; ACU11613; AEJ86603; AEJ86601; AEJ86599; AEJ86615; AEJ86604; AEJ86629; AEJ86606; AEJ86602; ACU11611; AFR90255; EMP38542; AAB03838; AFR90254; ACU45376; 2RCJ; 4Q9C; AYN64868; AYN64869; AES92988; AES92986; AES92959; AES92958; AES92984; AES92989; AES92987; UTD43068; BAJ20187; BAJ20186; AGG53233; AGG53242; AGG53246; AGG53244; AGG53234; AGG53260; ACZ65035; AGG53241; AGG53264; AGG53261; AGG53254; AAT02204.

<u>C_L domains accession codes:</u>

ACN09846; AWO67269; XP_047018211; XP_051739581; RXM94251; AWO67273; AWO67285; KAG8013225; KAG8013225; ACO13580; XP_050934017; ROI42561; ROL42386; ROL45405; ROL45405; ROL45405; TKS88155; KAG8003810; RXM36454; ACM09554; TKS80340; KAG8004842; ACO14165; ROI42554; KAG1948728; TKS80331; KAG8005388; KAG8005388; TWW70092; TWW52955; TWW52950; TWW70105; TKS80337; ROL45834; KAG1948731; ROL45835; TKS80333; TKS65975; ROL45837; KAG8000903; KAG8004815; KAI2646515; KAI2652986; KAG8015005; KAG8004848; KAG8004813; KAG8004844; KAF3707015; KAG1929366; KAG1929376; XP_017209378; KAF3707045; KAF3706995; KAF3706993; XP_049331886; XP_019209278; KAG8014350; KAI2652987; KAI2652987; 7PI2; AMN08822; XP_033024710; XP_028558593; XP_028607806; XP_031760076; XP_044151386;

XP_044151388; XP_029436932; 6XJA; PNI16580; AAD29610; AAB32987; AAD15786; BAC01684; BAC01702; AKL91152; QBK47591; BAC01749; BAC01680; BAC01723; BAC01672; BAC01713; BAC01707; BAC01737; BAC01692; BAC01709; BAC01732; BAC01676; AAA70229; BAC01752; BAC01698; BAC01694; BAC01724; BAC01699; BAC01722; BAC01714; BAC01704; BAC01695; QBK47577; BAC01690; AAC17968; AAB50880; 5VSI; XP_030864111; BAK48744; 6WG8; QJB76131; AFF60184; ATV91331; ATV91107; ATV91333; CAH17921; AAA18107; ACN96923; ACN96925; ACN96964; ACN96922; ACN96929; ACN96954; ACN96955; ACN96965; ACN96921; ACN96931; ACN96920; ACN96927; AAO84649; CAA53284; KAI5140899; AAA03520; P01835; AAO84653; ELK10654; ABV48920; AAA41405; KAG8512959; QJY40727; AAA41404; AAA41402; AAA39011; ABD64609; AAA39008; AAC37305; AAA41406; AAA41408; AAA39012; AAA39007; 6PYC; AAL17618; 6YHQ; KAI5939771; P01837; AAA38809; AWY11552; ABW24728; AAX47168; BAB90992; AAB53777; 1FPT; CZF87190; 3J42; XP_026908457; AAU45094; 7T0L; AAG43418; AAA41407; ABW24722; AAD40242; CAA24192; AAY83834; AAG43420; AAB53778; CAN87019; AAK01030; AAC98953; 1QFU; EHA99433; ABV02006; ABV02004; ATI97435; ATI97443; CAA38046; 3I50; AAF04610; AAA41403; AEM45012; AEM45004; ELR47093; ATI97444; EGW03655; KFO32621; AAA82732; KAH0519255; MBZ3873919; AAA31358; AAA31335; CCF72150; ELV10784; AAA51333; CAA88305; 6P62; KAB1256817; AAA31334; CAA10920; AAA31357; P01840; AAA31350; AAA31336; P03984; P01838; ABD64611; P01841; AAA31333; 7R8U; XP_048449493; XP_041035061; EMP26807; TFJ95003; XP_039353321; XP_049323828; KAI2648002; KAI2647426; KAI2647787; ROI42558; RXM98356; ROJ13904; KAI2648147; ROI42566; KAG8013240; XP_017315847; TMS15304; AWO67301; KAE8283257; TKS92024; XP_045066922; XP_021330477; TKS92025; ROL53049; TKS92023; KAE8283263; ACI66966; XP_021176414; XP_035035602; KAE8290125; ROL53045; XP_036797802; XP_045074771; KAG7504299; XP_014264359; KAF3700111; KAF3688195; XP_047199938; KAG8010901; KAG8010900; XP_034150846; XP_024658106; TSO37091; KAI2666591; KAI2646143; RXM95833; XP_047197646; XP_036006869; XP_036811263; KAF5883633; ROL44084; XP_024658107; KAG8010896; KAE8283260; TWW54037; TWW71864; KAF3706994; TWW54253; TWW53820; KAG8013218; TWW71862; KAG8013228; KAG8013229; KAG8013215; KAG8013236; KAG8013223; XP_024661989; XP_025759058; TWW53084; KAA0701453; XP_025756606; KAA0710206; XP_019212893; KAA0710207; KAA0710207; KAG1972221; AFA41931; XP_027669870; XP_050570205; KFQ94409; AMN08825; KFO05719; KFQ33173; KFU89639; KAF1663710; QAT77297; ARX27859; KFV96158; AMN08826; AMN08824; KFO13525; AMN08823; KFW81604; KFQ94410; P20763; AAA48906; XP_021405632; ACH44209; ACH44210; XP_035746355; XP_050565204; XP_015677573; XP_015677573; KAG8146258; KAG8146258; KAG8146257; KAG8146257; KAG8146257; KAG8146257; KAG8146259; KAG8146259; ETE68561; AAK07180; XP_033812901; XP_030043573; XP_033812642; XP_040261465; XP_030047891; XP_030045028; XP_033779923; XP_040180289; XP_029441631; AAO20093; AAO53356; P01843; AAO53357; AAO53417; AAO53418; AAO53416; AAA40096; AAO53347; P20766; AAA41419; XP_015442277; KAB1255245; CCN25613; AAO53393; AAR88376; KAI5929959; AAB24270; ELK15590; CAA40948; ABV22589; BAC01828; AAA59109; 4LLD; AAA58999; ABU90693; ABU90732; ABU90589; BAC01825; BAC01851; BAC01852; BAC01774; QJY40728; QJB76132; P0CF74; ATV91490; KAF6403527; XP_012997011; AAA30966; XP_023618956; QNT08900; ACN96934; 4LLM; ACN96935; 4LLQ; ACN96974; P01847; ACN96968; BAC01773; ACN96967; BAC01861; ABU90579; ASL06610; ABU90680; ACN96969; QBK47527; ABU90623; ANN81988; AEO16798; BAC01780; 4LLY; BAC01816; BAC01802; QCR98082; BAC01776; BAC01824; BAC01775; BAC01821; BAC01769; BAC01860; BAC01862; BAC01817; BAC01813; QCR98083; BAC01779; AAB28401; XP_012417851; ATV91493; ATV91491; KFO34801; XP_045220380; ACN96937; ACN96975; ACN96977; ACN96941; ACN96936; BAC01770; BAC01850; MBV95923; CAA27229; KAG8514505; CAA92268; AAB36578; ELK26785; EPQ11107; P01846; ELW49661; XP_044897840; ELK26787; ELK26786; AAA31360; AEM05840; AAA50983; XP_006768346; AAA31362; AAA31361; EGW07544; AIY24756; AEM05845; AEM05849; AAU45093; AIY23700; AEM05841; AET14246; AEM05838; AAK84156; AAA50971; AET14244; AET14243; AET14245; CAA27230; XP_004467769; QBK47507; KAH0512624; KAH0512624; ELR52172; AEM05834; AEM05851; AIY24744; AIY24804; AIY24755; AIY24754; AIY24757; AIY24813; AIY24821; ADK09717;

ADK09865; ADK09839; ADK09729; ADK09721; ADK09725; XP_028018162; XP_042090858; AIY24745; ADK09796; ADK09722; ADK09720; ADK09724; ADK09731; AAA31281; ELW68997; P01845; P01844; AAK01086; AAM76479; AAO16041; P20767; ADK09758; AIY23702; AET14252; AET14250; AIY24831; AIY24855; AAO16045; AAO16042; AAO53434; AAO53364; AAO53346; AAO53367; AET14248; AET14251; ADK09811; ADK09797; ADK09825; ADK09809; AIY24765; AIY24801; AIY24760; AIY24763; XP_014597487; ADK09714; ADK09768; ADK09763; ADK09854; ADK09833; ADK09726; ADK09826; ADK09841; ADK09757; AIY24766; AIY24845; AIY24856; ADK09829; AAO53435; ADK09708; AET14258; ELW68995; AAM76475; AAL37211; AAL37213; AAL37214; ABV02015; ABV02014; ABV02008; ABV02012; QWC93003; ABV02011; ABV02016; ABV02013; QWC93011; QWC93006; QWC93001; QWC93009; AAC98629; AAC98624; AAC98625; QWC93020; QWC93005; AAC98628; AAC98626; AAA49152; AAA49153; AAA86748; AAC59643; AAA86750; AAA50806; AAA86749; XP_025038876; XP_043385655; XP_043385656; XP_043385733; EMP41349; XP_043353699; EMP40248; EMP40248; XP_048677958; XP_032620624; XP_032620624; KAG6925585; XP_044842434; XP_032646324; XP_050774764; XP_023968828; XP_034643422; XP_037771953; XP_048676577; XP_025043132; XP_024058409; XP_030395824; XP_039355641;

	C_H1 domains						C_H3 domains						C_L domains		
	IgM	IgD/ IgW	IgA	IgG/ IgY	IgE	IgNA R	IgM	IgD/ IgW	IgA	IgG/ IgY	IgE	IgNA R	Ig*k*	Igλ	Total # of sequences per class
Mammalia	79	24	83	164	83	-	82	22	69	236	86	-	145	220	1293
Crocodylia	4	2	7	9	-	-	7	-	9	11	-	-	1	4	54
Aves	12	2	16	10	-	-	20	-	19	12	-	-	1	21	113
Testudines	4	10	-	8	-	-	5	6	-	9	-	-	3	23	68
Lepidosauria	5	2	2	5	-	-	8	2	-	5	-	-	3	11	43
Lissamphibia	5	4	-	7	-	-	8	2	-	7	-	-	4	10	47
Actinopterygii	44	8	-	-	-	-	46	9	-	-	-	-	59	71	237
Chondrichthyes	63	55	-	-	-	21	64	20	-	-	-	25	2	7	257
Total # of sequences per domain	738						789						585		

Table S1. Number of sequences in the dataset.

a)

C_H1 domain

```
                  A        AB      B          BC         C      CD      D           DE            E       EF   F          FG           G
                (1-15)           (16-26)    (27-38)   (39-45)        (77-84)                      (85-96)     (97-104)   (105-117)   (118-128)
            -------------->  ------------>            ------->      ------->                -------------> -------->              ------------
             1         10  15   16      2326 27     38 3941 45      77    84                 85  89     96  97    104 105        117 118
     87654321|........|....|123|......|..| |........| |.|...|1234567|......|12345677654321|...|......|12|......| |...........| |.........
5DK3      ......TKGPSVFPLAPCSRSTS...ESTAALGCLVK DYFP..EPVT VSWNSGALTS....GVHTFPAVLQSS......GLYSLSSVVTVPSSSL...GTKTYTC NVDHKP..SNTKV DKRV......
          |_____________________________________________________________________________________________________________________________|
IGHG4*01  ....ASTKGPSVFPLAPCSRSTS...ESTAALGCLVK DYFP..EPVT VSWNSGALTS....GVHTFPAVLQSS......GLYSLSSVVTVPSSSL...GTKTYTC NVDHKP..SNTKV DKRV
Homo sapiens
```

b)

C_H3 domain

```
                  A        AB      B          BC         C      CD      D           DE            E       EF   F          FG           G
                (1-15)           (16-26)    (27-38)   (39-45)        (77-84)                      (85-96)     (97-104)   (105-117)   (118-128)
            -------------->  ------------>            ------->      ------->                -------------> -------->              ------------
             1         10  15   16      2326 27     38 3941 45      77    84                 85  89     96  97    104 105        117 118
     87654321|........|....|123|......|..| |........| |.|...|1234567|......|12345677654321|...|......|12|......| |...........| |.........
3AVE      ......PREPQVYTLPPSRDELT...KNQVSLTCLVK GFYP..SDIA VEWESNGQPEN...NYKTTPPVLDSD......GSFFLYSKLTVDKSRW..QQGNVFSC SVMHEA.LHNHYT QKSLSLSP..
          |__________________________________________________________________________________________________________________________________|
IGHG1*01  ....GQPREPQVYTLPPSRDELT...KNQVSLTCLVK GFYP..SDIA VEWESNGQPEN...NYKTTPPVLDSD......GSFFLYSKLTVDKSRW..QQGNVFSC SVMHEA.LHNHYT QKSLSLSP
Homo sapiens
```

c)

C_L domain

```
                  A        AB      B          BC         C      CD      D           DE            E       EF   F          FG           G
                (1-15)           (16-26)    (27-38)   (39-45)        (77-84)                      (85-96)     (97-104)   (105-117)   (118-128)
            -------------->  ------------>            ------->      ------->                -------------> -------->              ------------
             1         10  15   16      2326 27     38 3941 45      77    84                 85  89     96  97    104 105        117 118
     87654321|........|....|123|......|..| |........| |.|...|1234567|......|12345677654321|...|......|12|......| |...........| |.........
5DK3      ........APSVFIFPPSDEQLK...SGTASVVCLLN NFYP..REAK VQWKVDNALQSG..NSQESVTEQDSKD.....STYSLSSTLTLSKADY..EKHKVYAC EVTHQG..LSSPV TKSFNRGE..
          |____________________________________________________________________________________________________________________________________|
IGKC*01   ....RTVAAPSVFIFPPSDEQLK...SGTASVVCLLN NFYP..REAK VQWKVDNALQSG..NSQESVTEQDSKD.....STYSLSSTLTLSKADY..EKHKVYAC EVTHQG..LSSPV TKSFNRGEC
Homo sapiens
```

Figure S1. IMGT numeration. **a**), **b**), **c**) C_H1, C_H3 and C_L IMGT alignment, respectively, including one representative structure for each domain. Above the alignment, the IMGT numeration and the corresponding secondary structure annotation. In pink, the residue corresponding to the conserved cysteines and in blue the conserved hydrophobic residues.

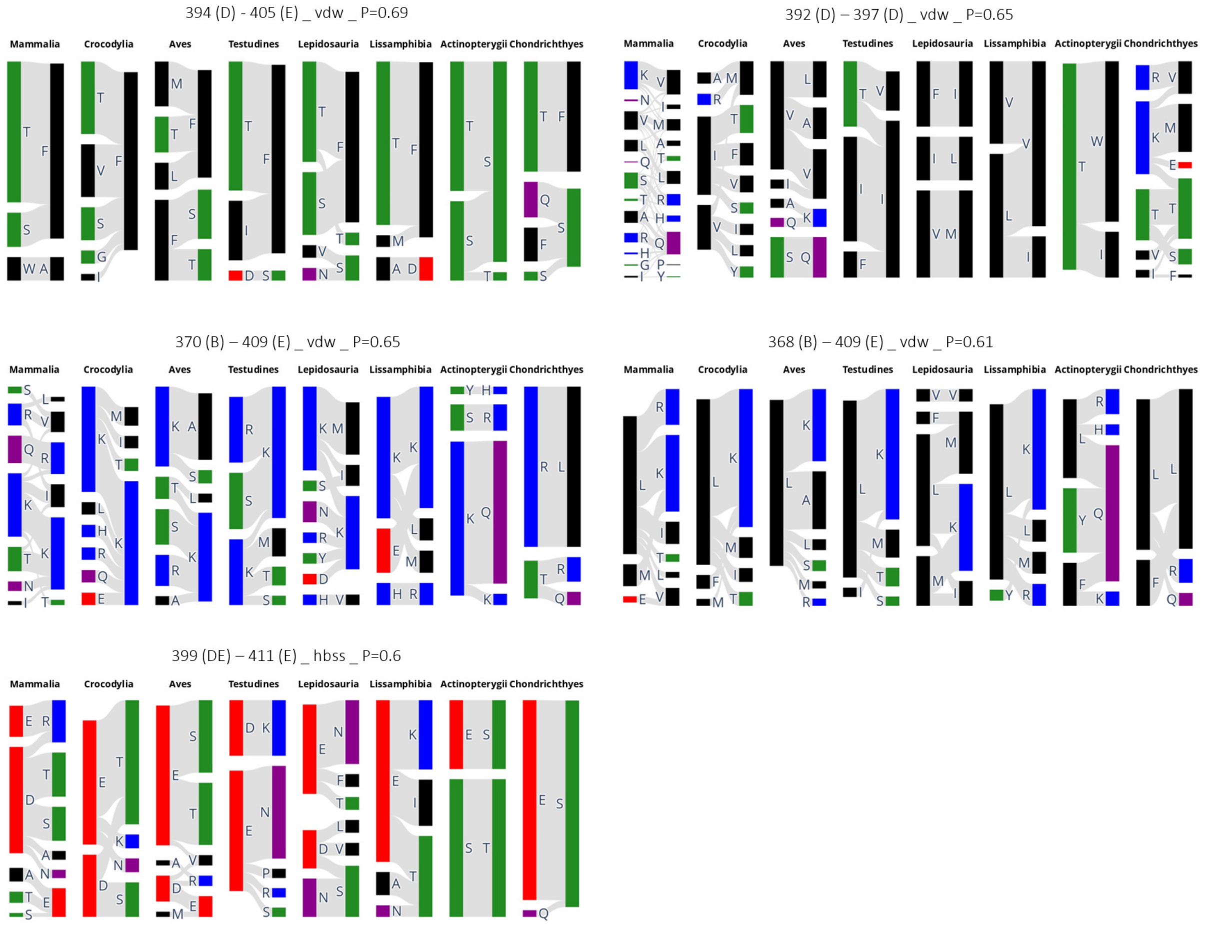

Figure S2. Evolutionary analysis of interdomain contacts. Plots showing the residue pairs present in the two coupled positions. The shown residue pairs are only those that occur in more than 2% of the total number of sequences within each animal class. The colors represent the biochemical property of the residue and are in line with the sequence logos color coding. The title of each plot includes the residue positions, the β-strand/loop where they are in the structure in brackets, the interaction type that the two residues make in the reference structure and the probability obtained from the EVcouplings analysis.

Residue's pointing direction	Average conservation scores
C_H2	0.22
Interdomain	0.25
Intradomain	0.31
Solvent	0.19

Table S2. Average conservation score. The conservation scores of the residues pointing in the same direction are averaged and the final value is shown here.

PDB	343	344	345	346	347	348	349	350	351	352	353	354	355	356	357	358	359	360	361	362	363	364	365	366	367
	P	R	E	P	Q	V	Y	T	L	P	P	S	R	D	E	L	T	K	N	Q	V	S	L	T	C

Alignment	1	2	3	4	5	6	7	8	9	10	11	12	13	14	15	16	17	18	19	20	21	22	23	24	25	26	27	28	29	30	31
	P	R	E	P	Q	V	-	-	-	-	Y	T	L	P	P	S	R	D	E	L	T	K	N	Q	V	-	-	S	L	T	C

IMGT	1.2	1.1	1	2	3	4	5	6	7	8	9	10	11	12	13	14	15	16	17	18	19	20	21	22	23
	P	R	E	P	Q	V	Y	T	L	P	P	S	R	D	E	L	T	K	N	Q	V	S	L	T	C

PDB	368	369	370	371	372	373	374	375	376	377	378	379	380	381	382	383	384	385	386	387	388	389	390	391	392
	L	V	K	G	F	Y	P	S	D	I	A	V	E	W	E	S	N	G	Q	P	E	N	N	Y	K

Alignment	32	33	34	35	36	37	38	39	40	41	42	43	44	45	46	47	48	49	50	51	52	53	54	55	56	57	58
	L	V	K	G	F	Y	P	S	D	I	A	V	E	W	E	S	N	G	Q	-	-	P	E	N	N	Y	K

IMGT	24	25	26	27	28	29	30	35	36	37	38	39	40	41	42	43	44	45	45.1	45.2	45.3	45.4	77	78	79
	L	V	K	G	F	Y	P	S	D	I	A	V	E	W	E	S	N	G	Q	P	E	N	N	Y	K

PDB	393	394	395	396	397	398	399	400	401	402	403	404	405	406	407	408	409	410	411	412	413	414	415	416	417
	T	T	P	P	V	L	D	S	D	G	S	F	F	L	Y	S	K	L	T	V	D	K	S	R	W

Alignment	59	60	61	62	63	64	65	66	67	68	69	70	71	72	73	74	75	76	77	78	79	80	81	82	83	84	85	86	87
	T	T	P	P	V	L	D	S	D	-	-	-	-	G	S	F	F	L	Y	S	K	L	T	V	D	K	S	R	W

IMGT	80	81	82	83	84	84.1	84.2	84.3	84.4	85.4	85.3	85.2	85.1	85	86	87	88	89	90	91	92	93	94	95	96
	T	T	P	P	V	L	D	S	D	G	S	F	F	L	Y	S	K	L	T	V	D	K	S	R	W

PDB	418	419	420	421	422	423	424	425	426	427	428	429	430	431	432	433	434	435	436	437	438	439	440	441	442	443	444	445
	Q	Q	G	N	V	F	S	C	S	V	M	H	E	A	L	H	N	H	Y	T	Q	K	S	L	S	P	G	K

Alignment	88	89	90	91	92	93	94	95	96	97	98	99	100	101	102	103	104	105	106	107	108	109	110	111	112	113	114	115	116	117
	Q	Q	G	N	V	F	S	C	S	V	M	H	E	A	L	H	N	H	Y	T	Q	K	S	L	S	L	S	P	G	K

IMGT	97	98	99	100	101	102	103	104	105	106	107	108	109	110	112	113	114	115	116	117	118	119	120	121	122	123	124
	Q	Q	G	N	V	F	S	C	S	V	M	H	E	A	L	H	N	H	Y	T	Q	K	S	L	S	L	S

Figure S3. Numeration. In the EVcouplings analysis, the numeration deriving from the CH3 domain of the reference PDB structure 3AVE is used (from 343 to 445). This numeration does not include gaps. In the alignment instead, we used a numeration starting from 1, which also consider gaps. Please note that, even if the sequences are the same, the length changes because the alignment includes gaps, that are instead missing in the reference PDB structure. In addition, we also provide the IMGT numeration for the same reference sequence. Please also note that, even if the C terminal residues PGK are included in the sequence alignment, they are not part of the domain, therefore they are missing in the IMGT alignment.

a)

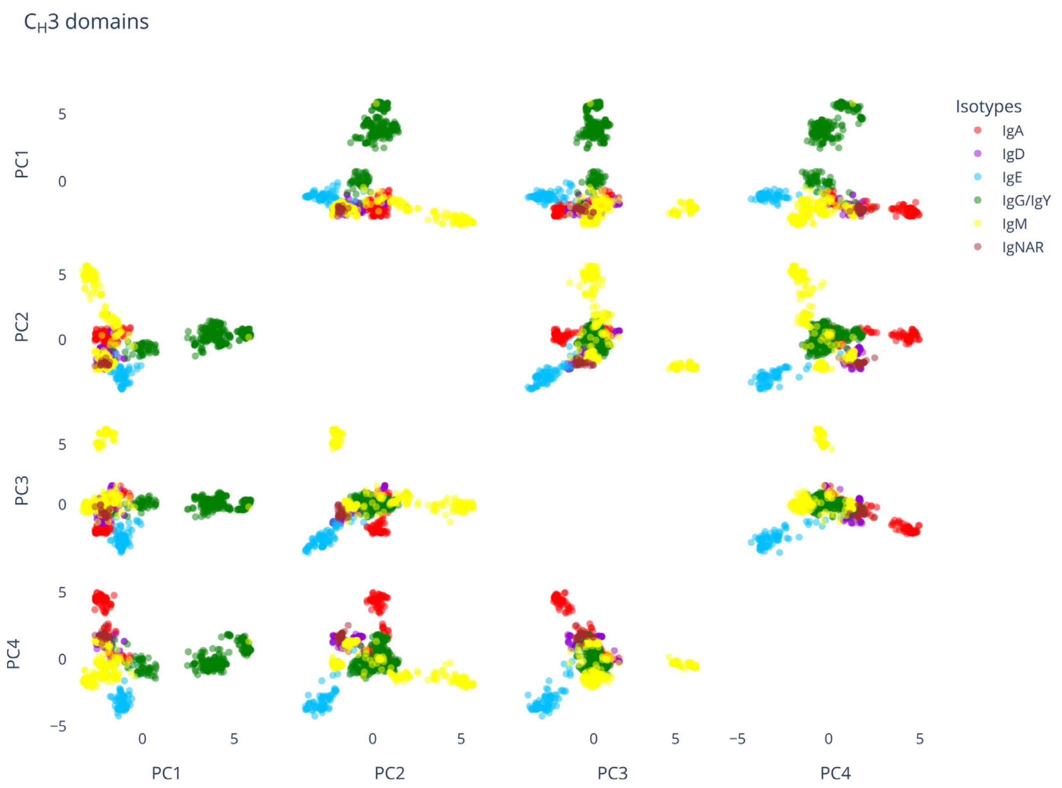
CH3 domains
Isotypes
IgA
IgD
IgE
IgG/IgY
IgM
IgNAR
PC1
PC2
PC3
PC4
5
0
−5
PC1
PC2
PC3
PC4

b)

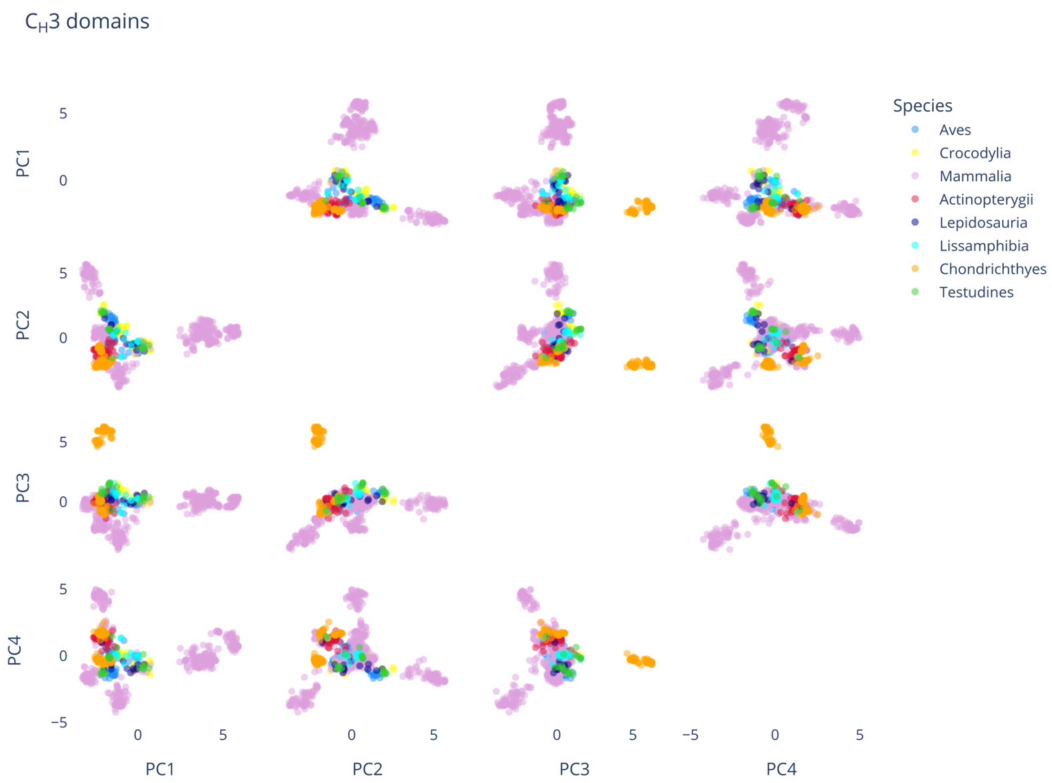
CH3 domains
Species
Aves
Crocodylia
Mammalia
Actinopterygii
Lepidosauria
Lissamphibia
Chondrichthyes
Testudines
PC1
PC2
PC3
PC4
5
0
−5
PC1
PC2
PC3
PC4

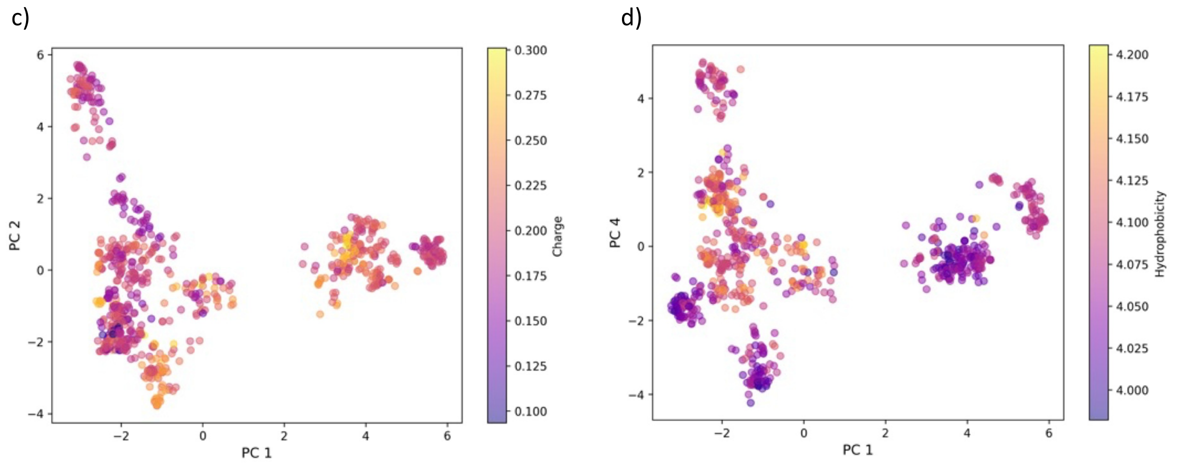

Figure S4. PCA analysis of the CH3 domains sequences. **a**) The PCA separation of the Ig isotype in the first four PCs space is shown. **b**) The same PCA space as in Figure S4a is colored according to the animal class that the sequence belongs to. **c**) Projection of the normalized charges in the PC1-PC2 plot. **d**) Projection of the normalized hydrophobicity value in the PC1-PC4 plot.

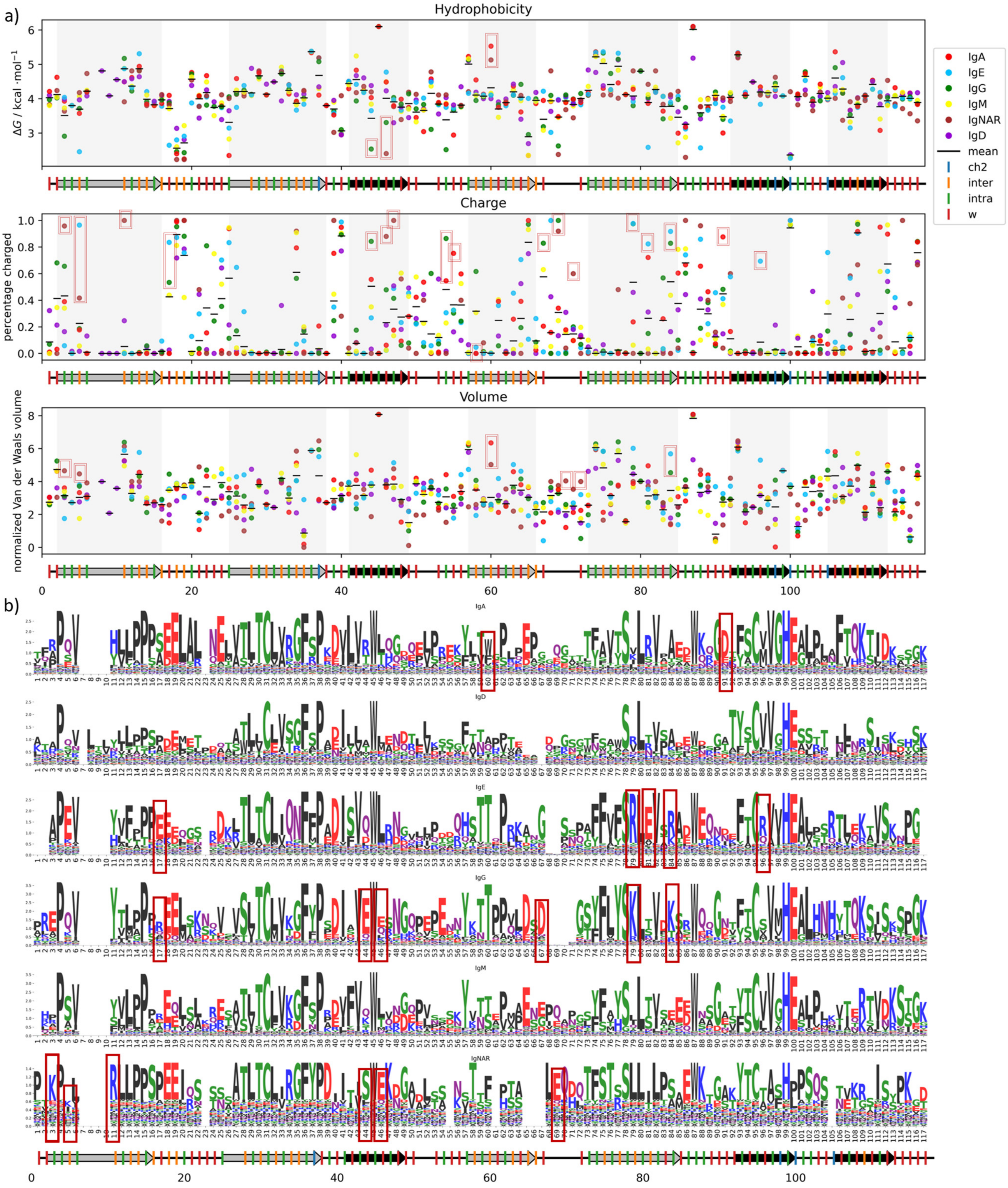

Figure S5. Biophysical properties of CH3 domains in different isotypes. **a)** Scatter plot showing the average value of hydrophobicity, charges and van der Waals volume, in each sequence position for each isotype. Below each subplot, a representation of the secondary structure of an exemplary CH3 domain: the light grey arrows represent the strands that point towards the facing domain, instead the black ones are the strands that point towards the solvent; the lines are the loops. The ticks on the secondary structure show where the residue points: if red, it points towards the solvent, green, in-between the β-sheets, orange, in the interface with the facing domain and blue, towards the CH2; a

missing tick means that the reference structure presents a gap in that position. In red squares the points that differ the most. **b)** Sequence logos of each isotype. The secondary structure is shown below. In red squares, the residues that differ the most.

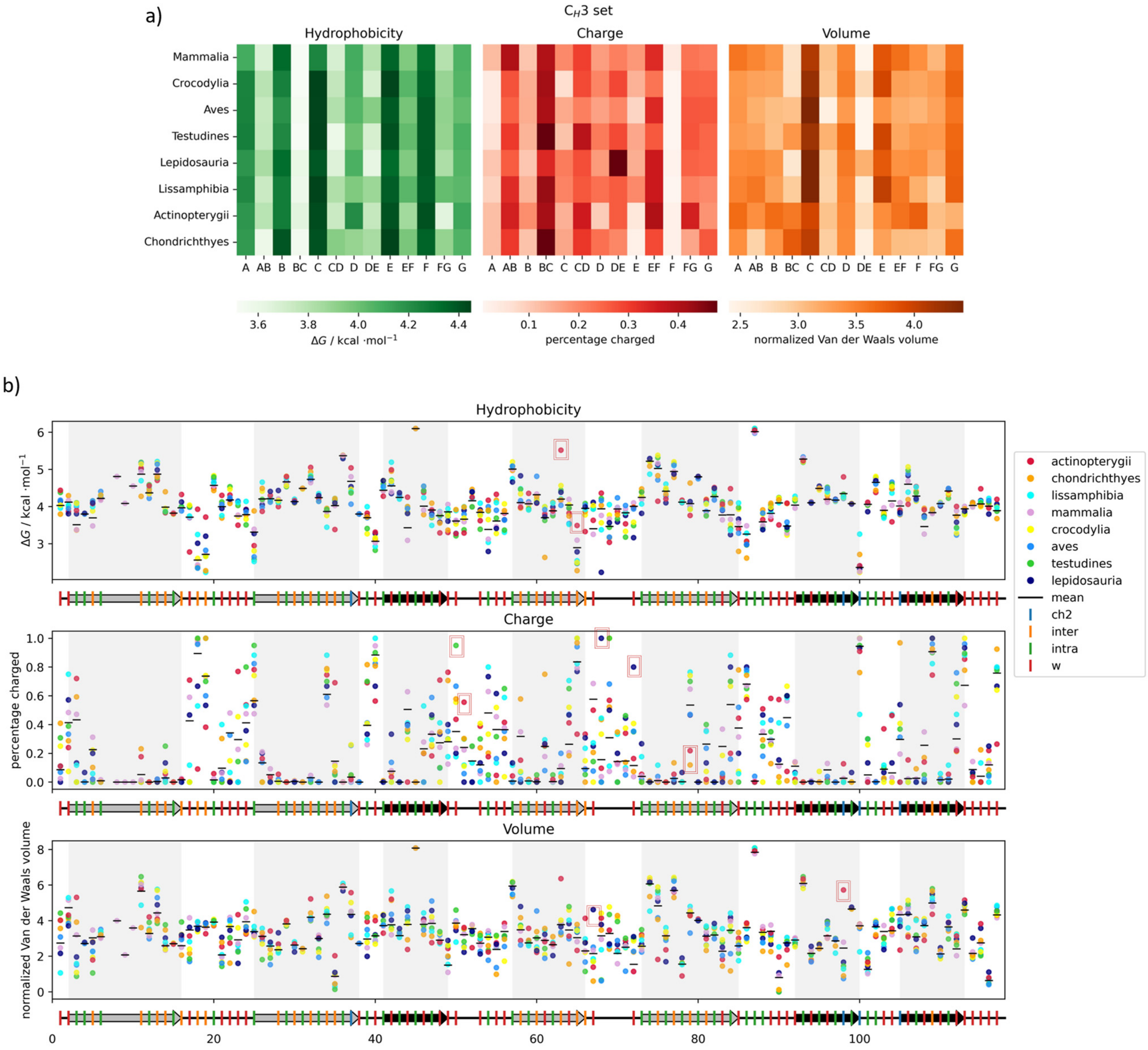

a)
C_H3 set
Hydrophobicity
Charge
Volume
Mammalia
Crocodylia
Aves
Testudines
Lepidosauria
Lissamphibia
Actinopterygii
Chondrichthyes
A AB B BC C CD D DE E EF F FG G
ΔG / kcal ·mol⁻¹
percentage charged
normalized Van der Waals volume
b)
Hydrophobicity
Charge
Volume
ΔG / kcal ·mol⁻¹
percentage charged
normalized Van der Waals volume
actinopterygii
chondrichthyes
lissamphibia
mammalia
crocodylia
aves
testudines
lepidosauria
mean
ch2
inter
intra
w

c)

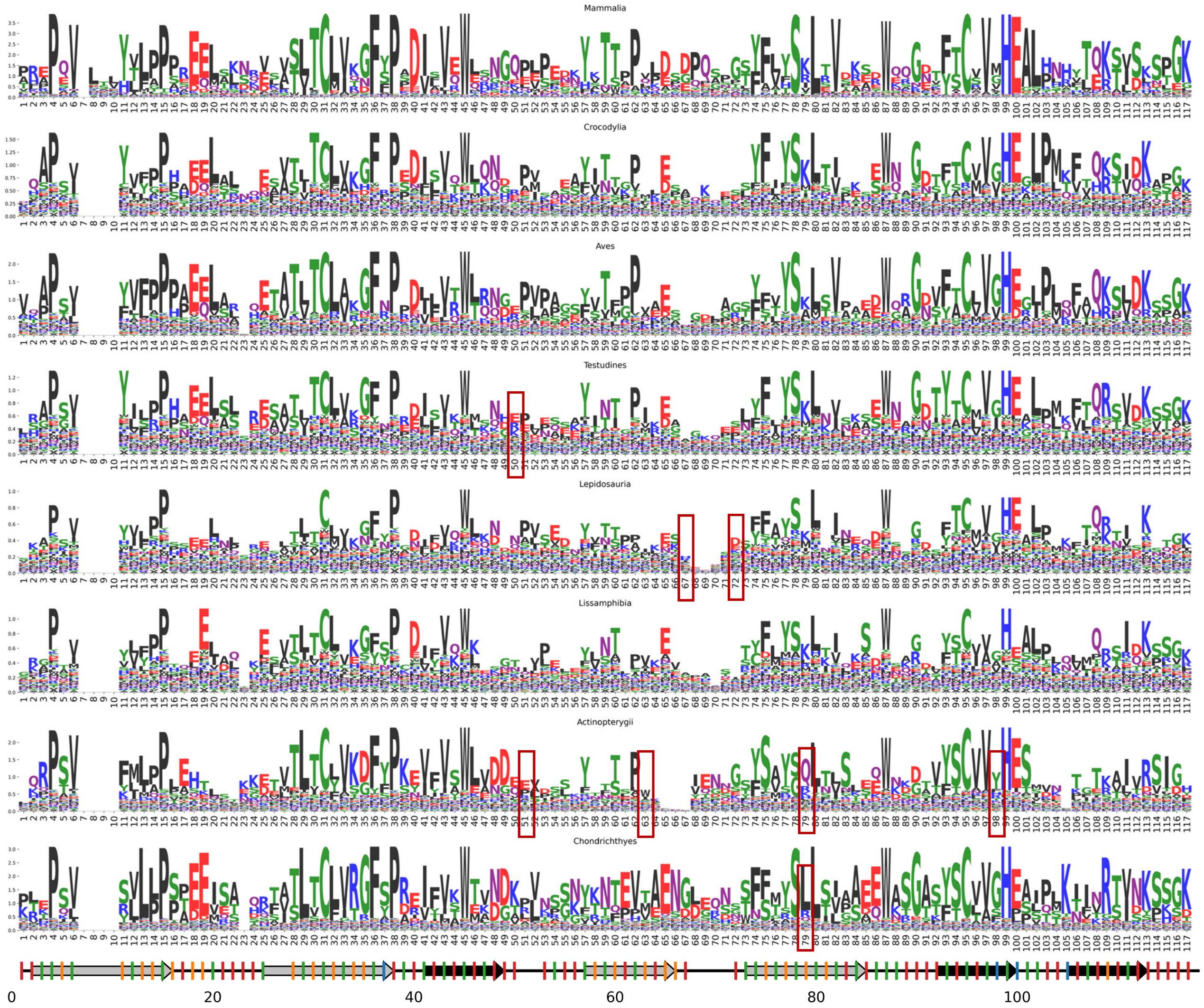

Figure S6. Biophysical properties of CH3 domains in different animal classes. **a**) Heatmap showing the occurrence of the biophysical property (hydrophobicity, charge and volume) in each secondary structure element for each animal class. **b**) Scatter plot showing the average value of hydrophobicity, charges and van der Waals volume, in each sequence position for each animal class. Below each subplot, a representation of the secondary structure of an exemplary CH3 domain: the light grey arrows represent the strands that point towards the facing domain, instead the black ones are the strands that point towards the solvent; the lines are the loops. The ticks on the secondary structure show where the residue points: if red, it points towards the solvent, green, in-between the β-sheets, orange, in the interface with the facing domain and blue, towards the CH2; a missing tick means that the reference structure presents a gap in that position. In red squares the points that differ the most. **c**) Sequence logos of each animal class. The secondary structure is shown below. In red squares, the residues that differ the most.

a)

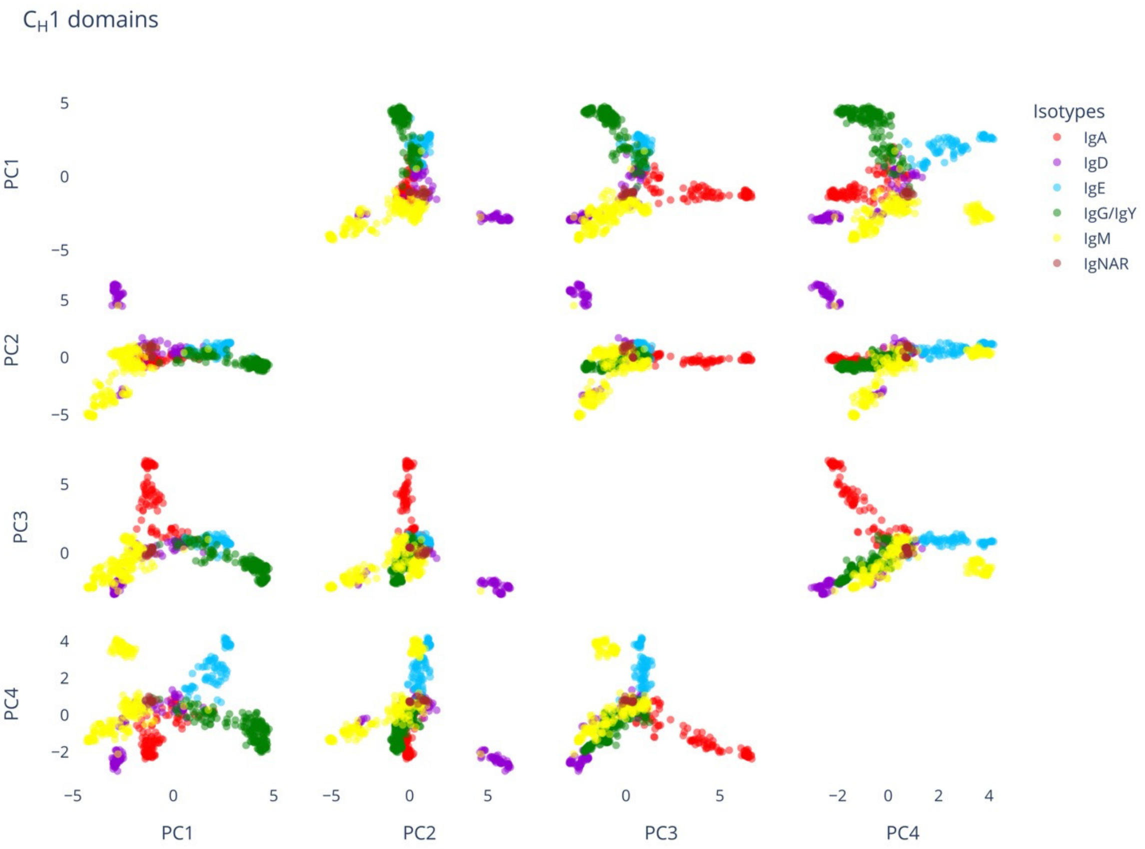

CH1 domains
PC1
PC2
PC3
PC4
Isotypes
IgA
IgD
IgE
IgG/IgY
IgM
IgNAR

b)

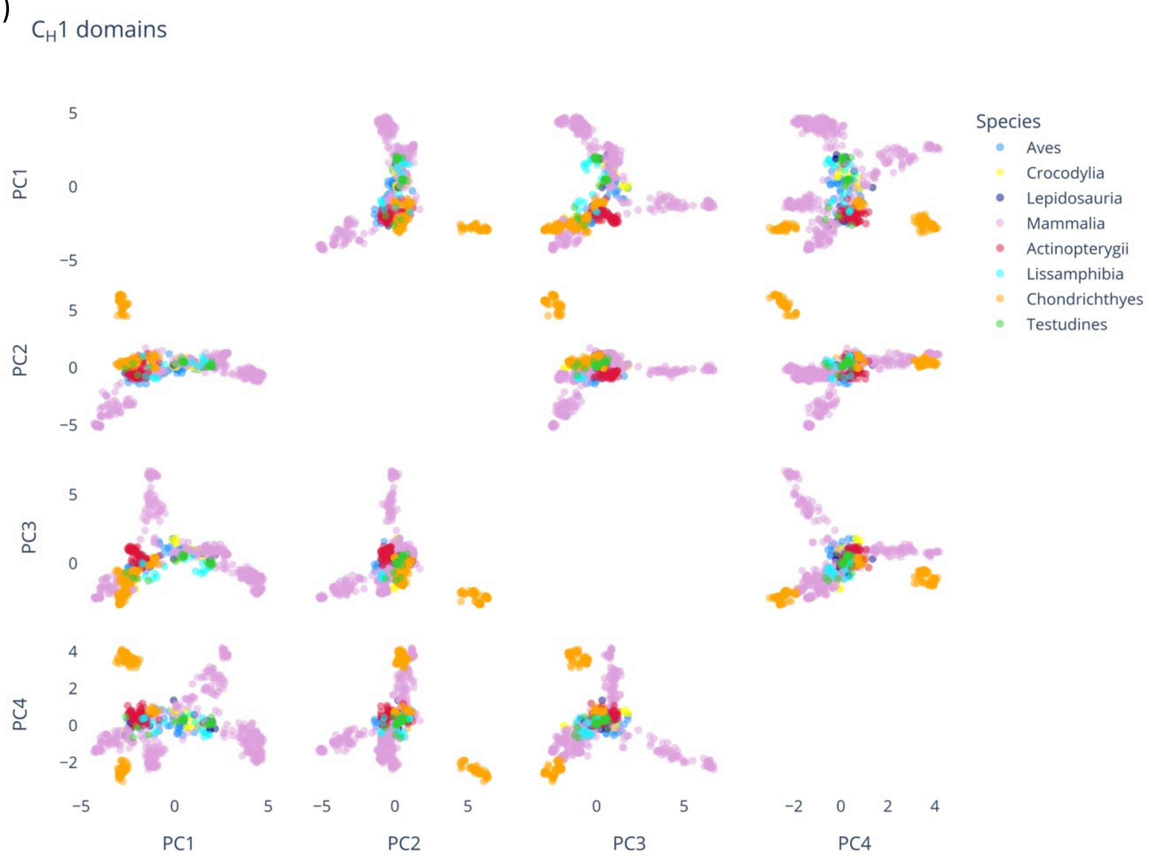

CH1 domains
PC1
PC2
PC3
PC4
Species
Aves
Crocodylia
Lepidosauria
Mammalia
Actinopterygii
Lissamphibia
Chondrichthyes
Testudines

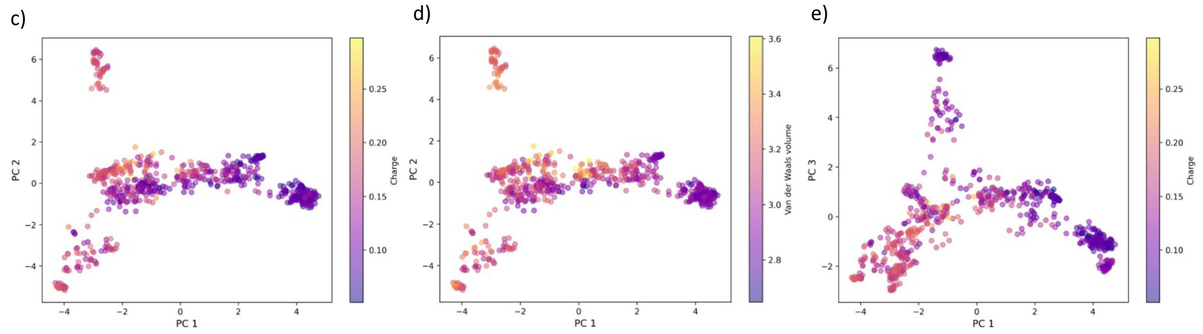

Figure S7. PCA analysis of the CH1 domains sequences. **a**) The PCA separation of the Ig isotype in the first four PCs space is shown. **b**) The same PCA space as in Figure S7a is shown, colored according to the animal class that the sequence belongs to. **c**) Projection of the normalized charges in the PC1-PC2 plot. **d**) Projection of the normalized van der Waals volume values in the PC1-PC2 plot. **e**) Projection of the normalized charges in the PC1-PC3 plot.

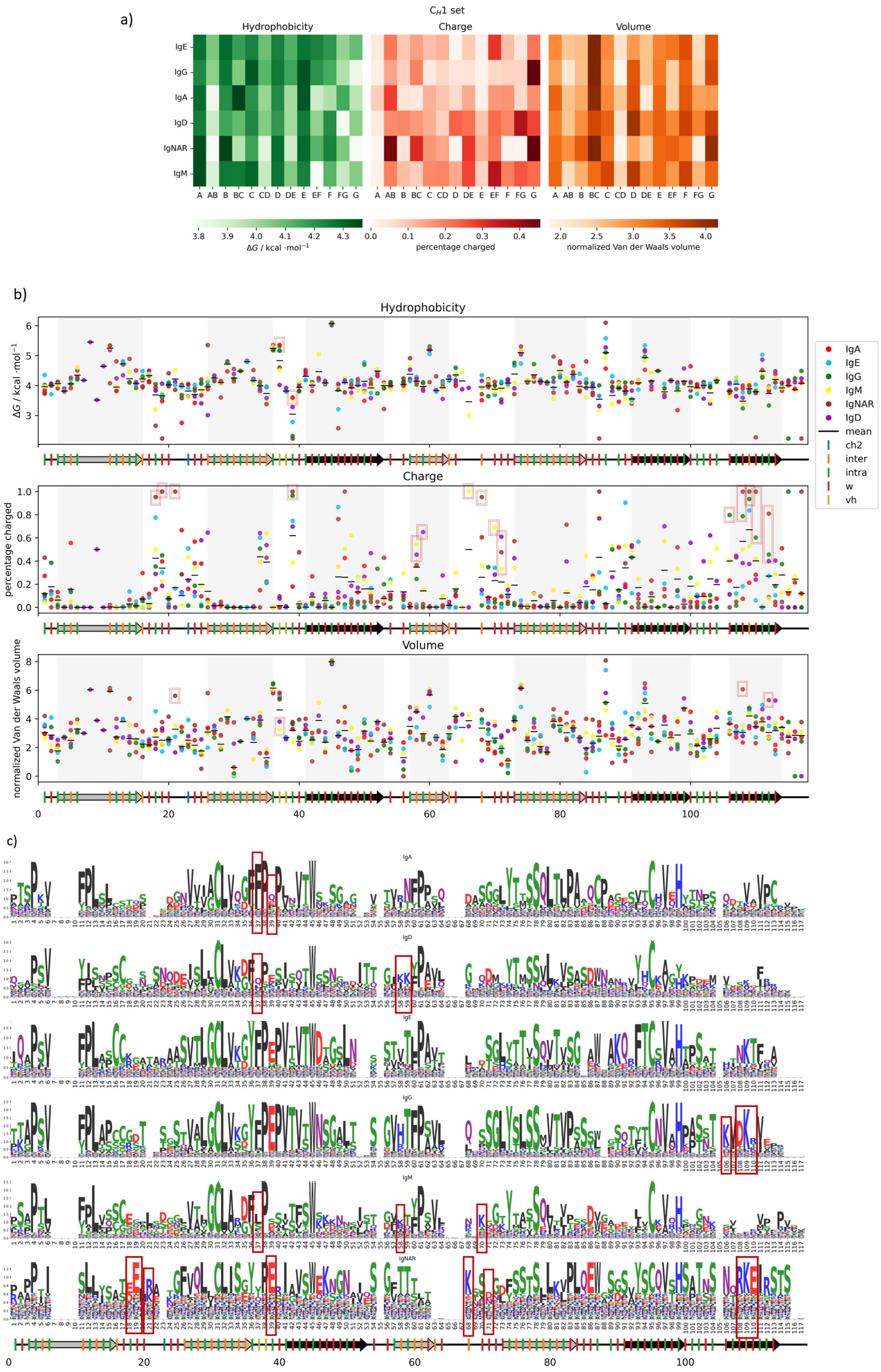

a)
C_H1 set
Hydrophobicity
Charge
Volume
IgE
IgG
IgA
IgD
IgNAR
IgM
A AB B BC C CD D DE E EF F FG G
3.8 3.9 4.0 4.1 4.2 4.3
ΔG / kcal ·mol^{-1}
0.0 0.1 0.2 0.3 0.4
percentage charged
2.0 2.5 3.0 3.5 4.0
normalized Van der Waals volume
b)
Hydrophobicity
ΔG / kcal ·mol^{-1}
Charge
percentage charged
Volume
normalized Van der Waals volume
IgA
IgE
IgG
IgM
IgNAR
IgD
mean
ch2
inter
intra
w
vh
0 20 40 60 80 100
c)
IgA
IgD
IgE
IgG
IgM
IgNAR
0 20 40 60 80 100

Figure S8. Biophysical properties of CH1 domains in different isotypes. **a**) Heatmap showing the occurrence of the biophysical property (hydrophobicity, charge and volume) in each secondary structure element for each Ig isotype. **b**) Scatter plot showing the average value of hydrophobicity, charges and van der Waals volume, in each sequence position for each Ig isotype. Below each subplot, a representation of the secondary structure of an exemplary CH1 domain: the light grey arrows represent the strands that point towards the facing domain, instead the black ones are the strands that point towards the solvent; the lines are the loops. The ticks on the secondary structure show where the residue points: if red, it points towards the solvent, green, in-between the β-sheets, orange, in the interface with the facing domain, blue, towards the CH2, and yellow towards the variable domain; a missing tick means that the reference structure presents a gap in that position. In red squares the points that differ the most. **c**) Sequence logos of each Ig isotype. The secondary structure is shown below. In red squares, the residues that differ the most.

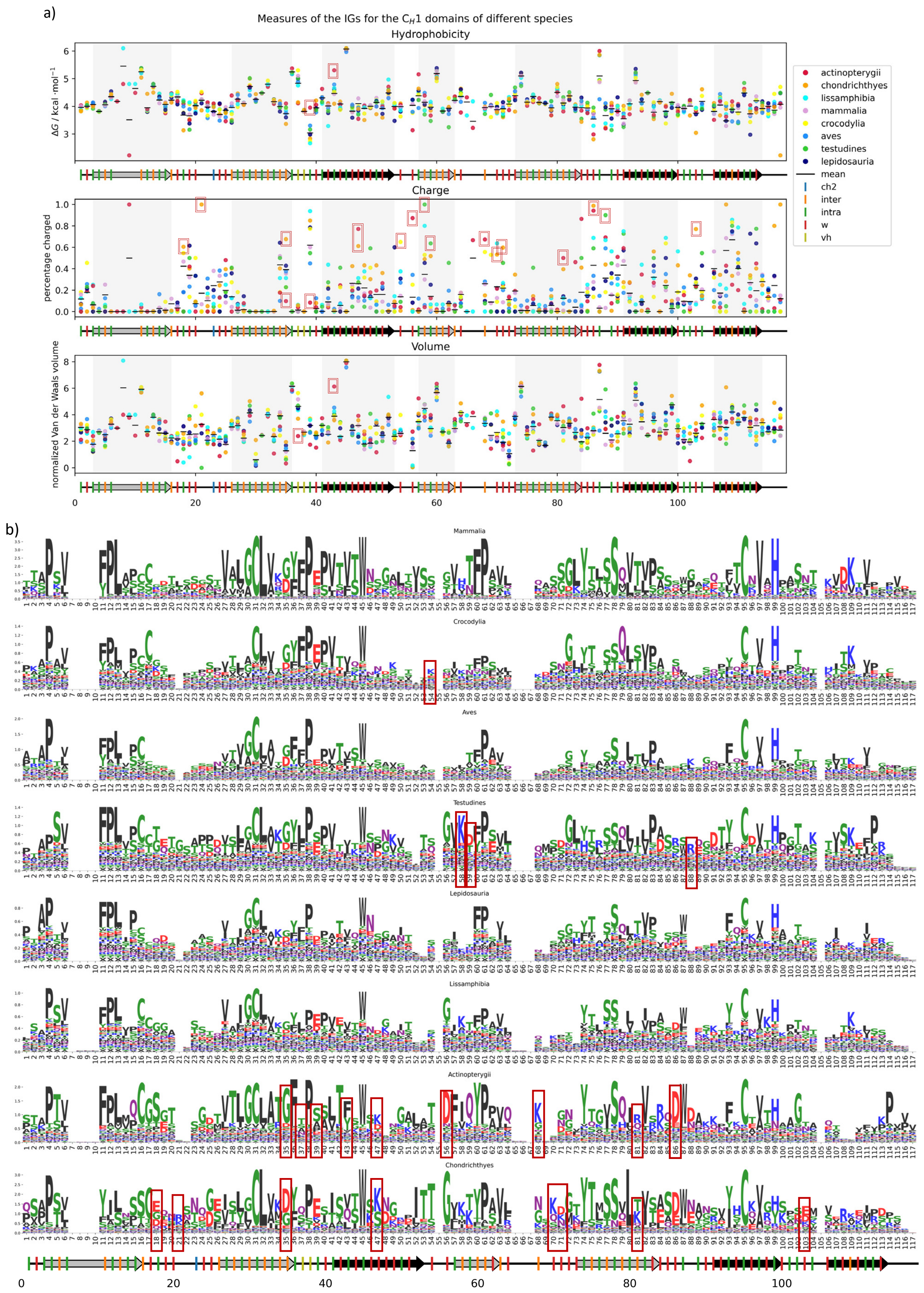

a)
Measures of the IGs for the CH1 domains of different species
Hydrophobicity
ΔG / kcal ·mol⁻¹
Charge
percentage charged
Volume
normalized Van der Waals volume
actinopterygii
chondrichthyes
lissamphibia
mammalia
crocodylia
aves
testudines
lepidosauria
mean
ch2
inter
intra
w
vh
b)
Mammalia
Crocodylia
Aves
Testudines
Lepidosauria
Lissamphibia
Actinopterygii
Chondrichthyes

Figure S9. Biophysical properties of CH1 domains in different animal classes. **a**) Scatter plot showing the average value of hydrophobicity, charges and van der Waals volume, in each sequence position for each animal class. Below each subplot, a representation of the secondary structure of an exemplary CH1 domain: the light grey arrows represent the strands that point towards the facing domain, instead the black ones are the strands that point towards the solvent; the lines are the loops. The ticks on the secondary structure show where the residue points: if red, it points towards the solvent, green, in-between the β-sheets, orange, in the interface with the facing domain, blue, towards the CH2, and yellow towards the variable domain; a missing tick means that the reference structure presents a gap in that position. In red squares the properties that differ the most. **b**) Sequence logos of CH1 domain in each animal class. The secondary structure is shown below. In red squares, the residues that differ the most.

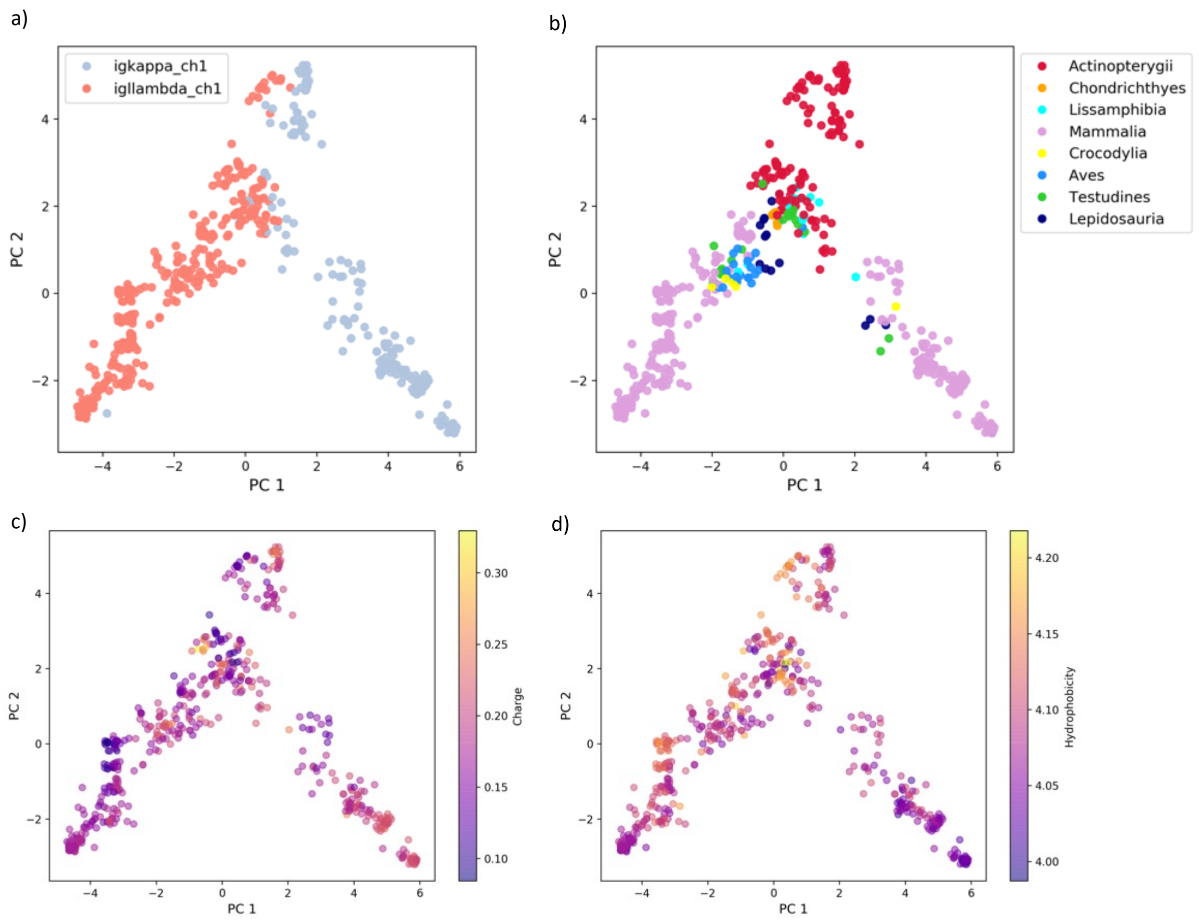

Figure S10. PCA analysis of the CL domains sequences. **a**) The PCA separation of the Ig light chain isotypes. **b**) The same PCA space as in Figure S10a is shown, colored according to the animal class that the sequence belongs to. **c**) Projection of the normalized charges in the PC1-PC2 plot. **d**) Projection of the normalized hydrophobicity values in the PC1-PC2 plot.

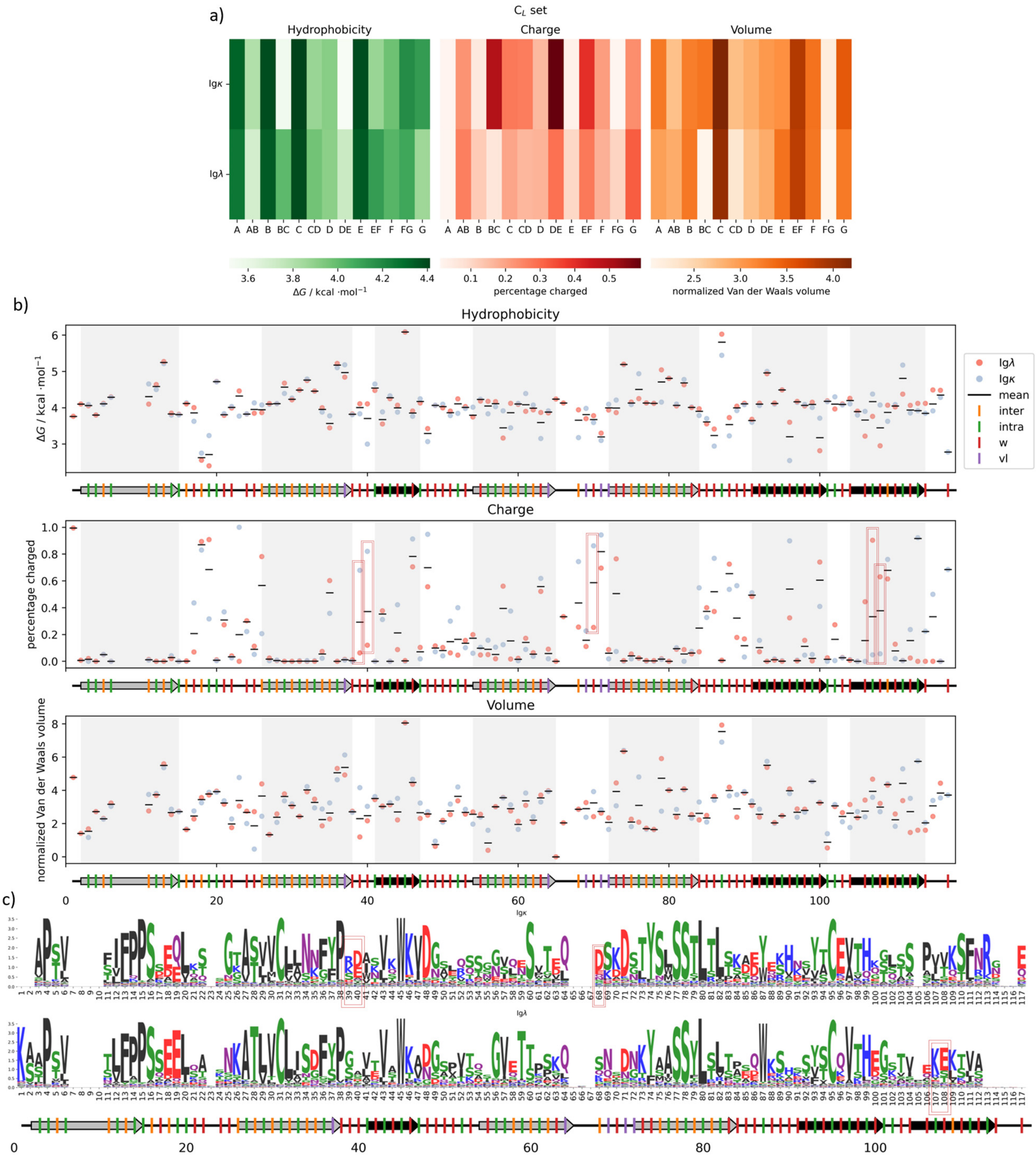

Figure S11. Biophysical properties of CL domains in different isotypes. **a**) Heatmap showing the occurrence of the biophysical property (hydrophobicity, charge and volume) in each secondary structure element for each Ig light chain isotype. **b**) Scatter plot showing the average value of hydrophobicity, charges and van der Waals volume, in each sequence position for each Ig light chain isotype. Below each subplot, a representation of the secondary structure of an exemplary CL domain (PDB: 5DK3): the light grey arrows represent the strands that point towards the facing domain, instead the black ones are the strands that point towards the solvent; the lines are the loops. The ticks on the secondary structure show where the residue points: if red, it points towards the solvent, green, in-between the β-sheets, orange, in the interface with the facing domain, and purple towards the variable domain; a missing tick means that the reference structure presents a gap in that position. In red squares the point that differ the most. **c**) Sequence logos of each Ig light chain isotype. The secondary structure is shown below. In red squares, the residues that differ the most.

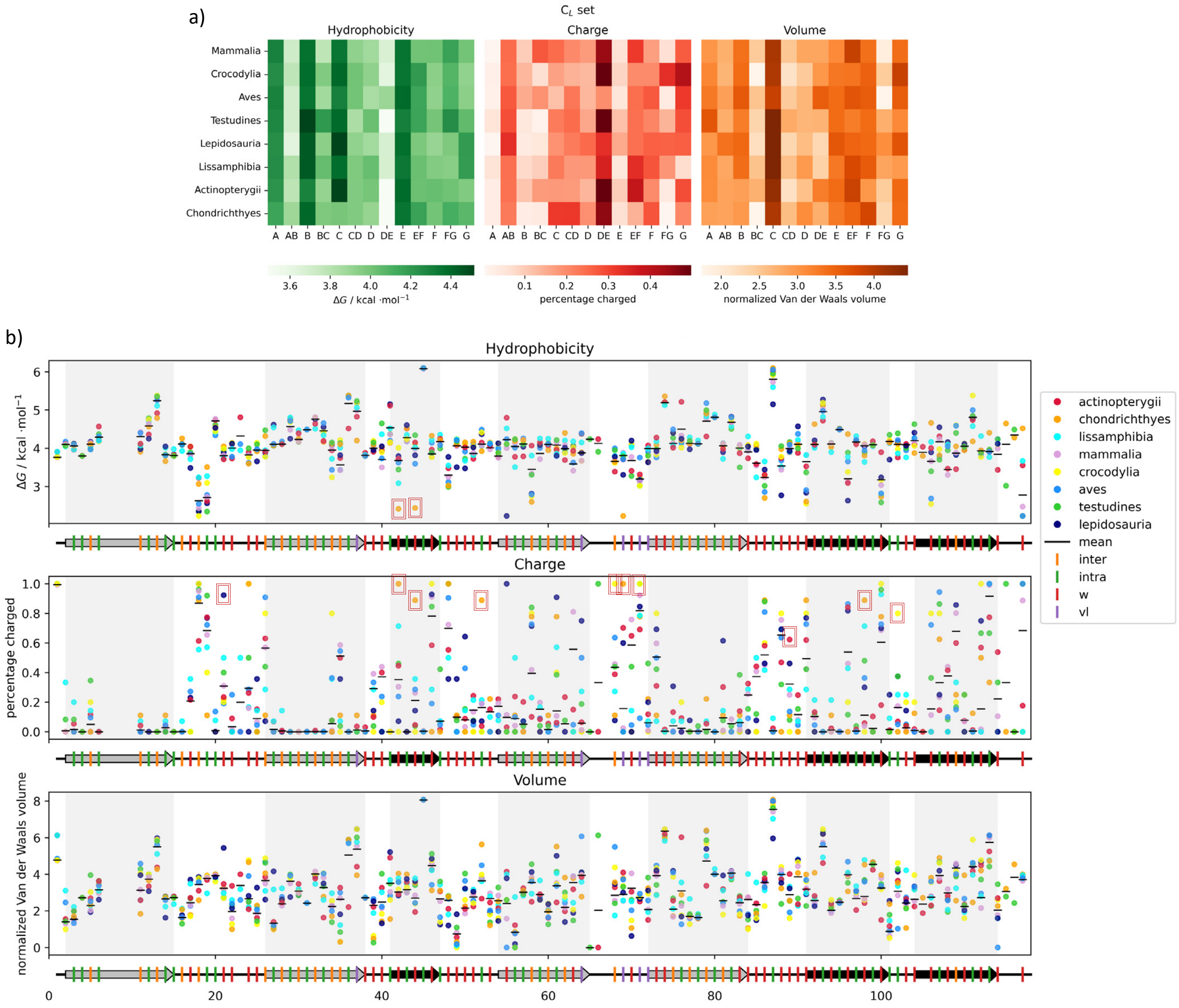

a)
C_L set
Hydrophobicity
Charge
Volume
Mammalia
Crocodylia
Aves
Testudines
Lepidosauria
Lissamphibia
Actinopterygii
Chondrichthyes
A AB B BC C CD D DE E EF F FG G
3.6 3.8 4.0 4.2 4.4
ΔG / kcal ·mol⁻¹
0.1 0.2 0.3 0.4
percentage charged
2.0 2.5 3.0 3.5 4.0
normalized Van der Waals volume
b)
Hydrophobicity
ΔG / kcal ·mol⁻¹
Charge
percentage charged
Volume
normalized Van der Waals volume
0 20 40 60 80 100
actinopterygii
chondrichthyes
lissamphibia
mammalia
crocodylia
aves
testudines
lepidosauria
mean
inter
intra
w
vl

c)

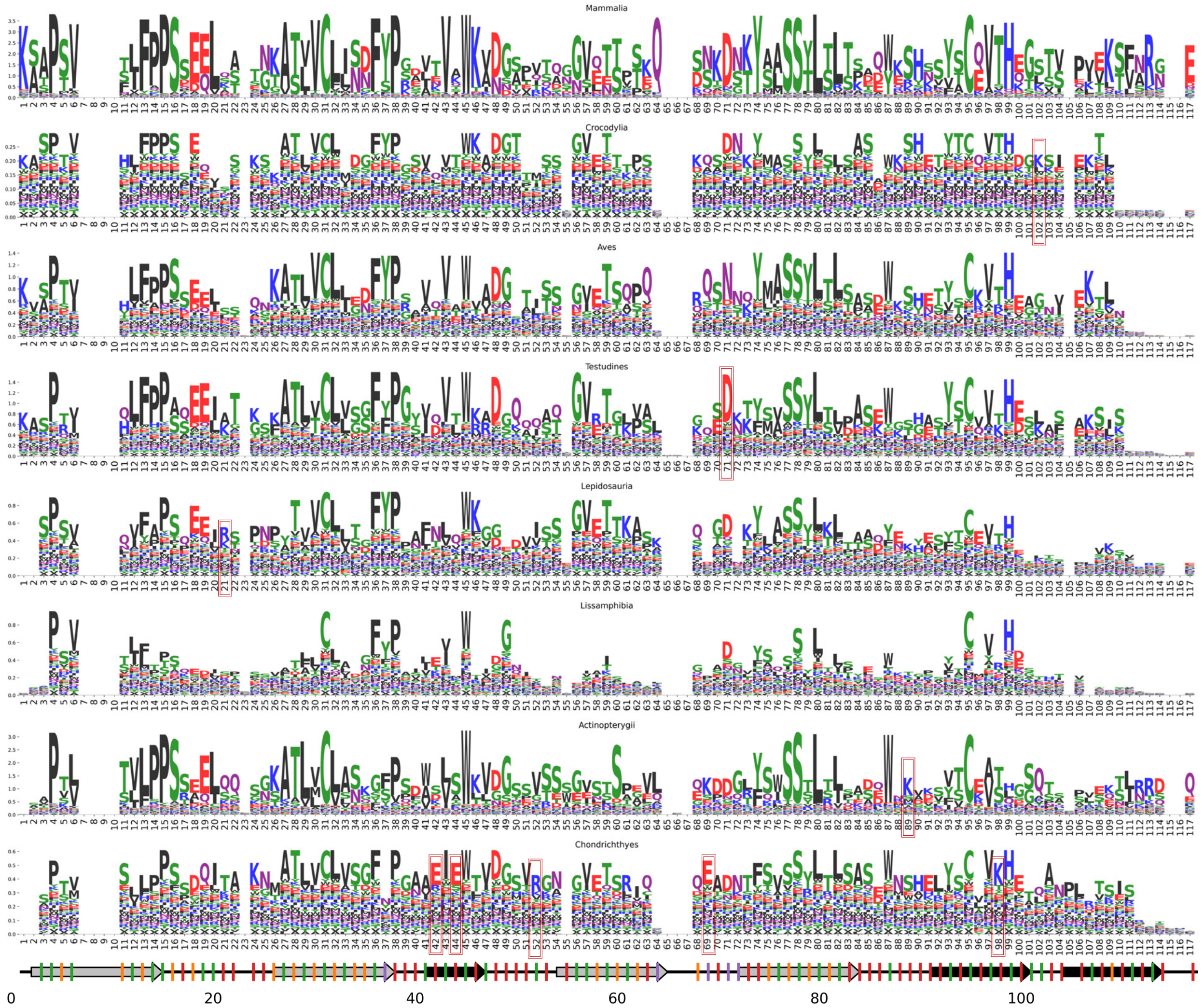

Figure S12. Biophysical properties of CL domains in different animal classes. **a**) Heatmap showing the occurrence of the biophysical property (hydrophobicity, charge and volume) in each secondary structure element for each animal class. **b**) Scatter plot showing the average value of hydrophobicity, charges and van der Waals volume, in each CL domain sequence position for each animal class. Below each subplot, a representation of the secondary structure of an exemplary CL domain: the light grey arrows represent the strands that point towards the facing domain, instead the black ones are the strands that point towards the solvent; the lines are the loops. The ticks on the secondary structure show where the residue points: if red, it points towards the solvent, green, in-between the β-sheets, orange, in the interface with the facing domain, and purple towards the variable domain; a missing tick means that the reference structure presents a gap in that position. In red squares the points that differ the most. **c**) Sequence logos of CL domain alignments for each animal class. The secondary structure is shown below. In red squares, the residues that differ the most.
